# Induction of hemagglutinin stalk reactive antibodies by the administration of a live-attenuated influenza virus vaccine in children

**DOI:** 10.1016/j.isci.2025.112893

**Published:** 2025-06-13

**Authors:** Juan Manuel Carreño, Philip Meade, Na Fatimata Sogodogo, Kaori Sano, Johnstone Tcheou, Ariel Raskin, Gagandeep Singh, Miriam Fried, Madhumathi Loganathan, Benjamin Francis, Dominika Bielak, Ya Jankey Jagne, Hadijatou J. Salah, Florian Krammer, Thushan I. de Silva

**Affiliations:** 1 Department of Microbiology, Icahn School of Medicine at Mount Sinai, New York, NY, USA; 2 Center for Vaccine Research and Pandemic Preparedness (C-VARPP), Icahn School of Medicine at Mount Sinai, New York, NY, USA; 3 Vaccines and Immunity Theme, Medical Research Council Unit The Gambia at the London School of Hygiene & Tropical Medicine, Banjul, The Gambia; 4 Department of Pathology, Molecular and Cell Based Medicine, Icahn School of Medicine at Mount Sinai, New York, NY, USA; 5 Ignaz Semmelweis Institute, Interuniversity Institute for Infection Research, Medical University of Vienna, Vienna, Austria; 6 Division of Clinical Medicine, School of Medicine and Population Health, University of Sheffield, Sheffield, UK; 7 The Florey Institute of Infection and Sheffield NIHR Biomedical Research Centre, The University of Sheffield, Sheffield, UK

**Keywords:** Immunity

## Abstract

Early life exposures to influenza viruses may imprint a hemagglutinin group-specific signature on immunity that impacts future responses to infection or vaccination. We assessed the administration of a live attenuated influenza virus (LAIV) vaccine in children. Two LAIV formulations (2016–17 and 2017–18) containing distinct H1N1 components were used. Modest boosting of pre-existing serum stalk reactive titers and enhancement of functional antibody-dependent cellular cytotoxicity activity (ADCC) was observed. The magnitude of stalk antibody induction in children naive to influenza A viruses was low; however, LAIV induced *de novo* stalk antibodies, increasing the number of children seropositive to both group 1 (G1) and group 2 (G2) influenza viruses. The 2018 LAIV formulation, containing an updated H1N1 component, induced higher stalk reactive antibodies with strong ADCC effector functions to the G1 stalk. No significant changes were detected in NA-reactive antibodies in serum or in stalk- or NA-secretory IgA (sIgA) in oral fluid.

## Highlights

• LAIV can induce *de novo* group and 1 and 2 anti-stalk antibodies in children
• LAIV can boost pre-existing stalk antibodies and increase ADCC activity
• Overall increase in anti-stalk antibody levels is modest
• 2017-18 LAIV formulation induced higher anti-stalk titers in group 1

## Graphical abstract

**Figure:**
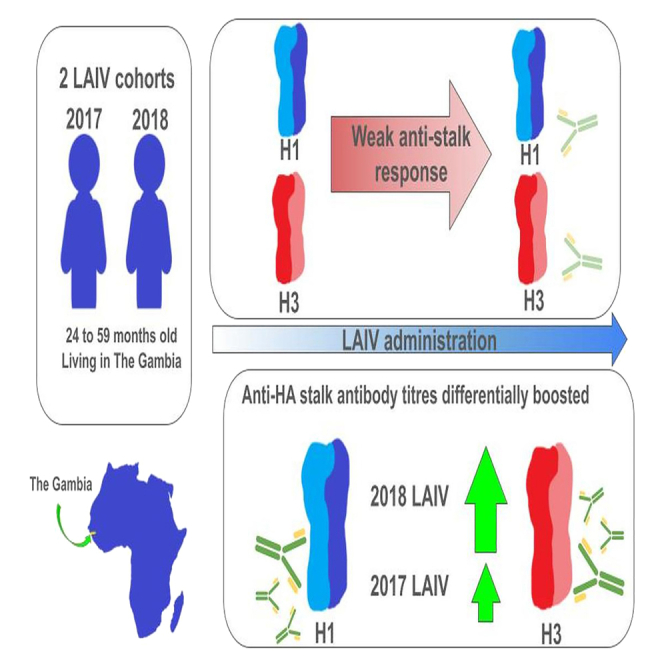

## Introduction

The wide variety of antigenically distant influenza A virus subtypes cluster in two major groups based on the phylogeny of the hemagglutinin (HA) gene: group 1 and group 2.^1^ While the HA head is highly variable, HA stalk regions are more conserved within each group. Emerging data suggest that the first exposure(s) in life to representative subtypes from either of these groups may enhance future immunological responses to influenza A viruses from the same group.^2^^,^^3^^,^^4^^,^^5^ Individuals initially exposed to H1N1 (group 1) have been found to be better protected from developing severe disease caused by zoonotic H5N1 (group 1) viruses, but lack similar protection from severe disease caused by H7N9 (group 2).^2^ Likewise, individuals initially exposed to H3N2 (group 2), who are better protected against H7N9, lacked enhanced protection to H5N1.^2^ A similar effect has been observed during future exposures to other seasonal influenza viruses.^5^ This immunological priming or imprinting effect, established early in life, appears to prevail over a lifetime. This phenomenon is consistent with immunological memory and is thought to impact not only future influenza virus infections but responses to vaccination as well. This imprinting effect is thought to be mediated in part by antibodies against the more conserved HA stalk region, which have also been shown to be an independent correlate of protection in community-based cohort studies.^6^ Moreover, it has been hypothesized that modulating these responses very early in life through vaccination would permit equal imprinting of the population against group 1 and group 2 influenza viruses, to avoid skewed responses toward a certain phylogenetic group later in life, and allow for enhanced protection against both groups.^7^ This could also have a positive effect if these imprinted responses are later subject to targeted boosting with universal influenza virus vaccine candidates such as the HA stalk-based vaccines.^8^^,^^9^^,^^10^

Potentially, ‘equivalent imprinting’ across both group 1 and 2 stalk antigens could be achieved with multicomponent inactivated virus or recombinant protein-based vaccines delivered in early life. However, an alternative to induce not only systemic immunity, but also robust T and B cell responses at mucosal surfaces of the upper respiratory tract, would be through the administration of live attenuated influenza virus vaccines (LAIVs). Intranasally administered LAIVs are safe and immunogenic in humans^11^ and can elicit influenza virus specific T and B cells in the upper respiratory tract,^12^ as well as mucosal antibodies.^13^^,^^14^ These responses are desirable to block virus infection and prevent virus transmission to susceptible hosts. Importantly, LAIV is the preferred vaccine for many influenza vaccination programs in children aged two years onwards. However, like other vaccine types, LAIV elicits limited mucosal, neuraminidase- and stalk-reactive antibodies in adults.^15^ Limited data are available on the ability of LAIV to induce stalk antibodies in very young children. Hence, we sought to determine if these types of responses could be induced through the administration of LAIV to influenza vaccine naive young children, with different influenza virus exposure histories. We used samples from children 24–59 months of age in The Gambia who were vaccinated with a WHO pre-qualified Leningrad-backbone trivalent LAIV containing either an A/17/California/2009/38 (Cal09) or A/17/New York/15/5364 (NY15) H1N1 component. We measured neuraminidase- and stalk-reactive antibodies in serum and secretory IgA (sIgA) antibodies in oral fluid.

We found that LAIV was able to boost pre-existing stalk reactive antibody titers, with an associated increase in antibody dependent cellular cytotoxicity (ADCC) activity. While these LAIV-driven increases were modest compared to those induced by prior influenza virus exposure, higher induction was seen in children with lower pre-vaccination titers. LAIV was able to induce *de novo* stalk reactive antibodies to increase the proportion of children who were seropositive to both group 1 and 2 following vaccination. LAIV containing the NY15 pH1N1 resulted in higher group 1 stalk antibody induction and boosting. Our results add to existing data about the antibody response following LAIV administration to young children.

## Results

### Changes in hemagglutinin-specific antibody titers and antibody breadth in young children after live attenuated influenza virus administration

Repeated exposures to influenza viruses can induce an increase in the magnitude and breadth of the antibody responses over time. However, the synchronicity and specificity of these responses during the very first exposures early in life and the impact of these variables on vaccination at an early age remain unclear. To understand how children of different ages and with different influenza virus exposure histories respond to the administration of LAIV, we used samples from an open-label, observational, phase 4 study in which children aged 24–59 months at Sukuta, a periurban area in The Gambia.^16^ All children were influenza vaccine naive. Children received one dose of the WHO pre-qualified Leningrad-backbone^17^ trivalent LAIV containing either A/17/California/2009/38 (Cal09 pH1N1), A/17/Hong Kong/2014/8296 (H3N2), and B/Texas/02/2013 (B/Victoria/2/87-like lineage) or A/17/New York/15/5364 (NY15 pH1N1), A/17/Hong Kong/2014/8296 (H3N2), and B/Texas/02/2013 (B/Victoria/2/87-like lineage), depending on the year of enrollment. 118 children received one dose of the Cal09 LAIV from 2016 to 2017, and a different cohort of 126 children received one dose of the NY15 LAIV from 2017 to 2018.

**Figure 1:**
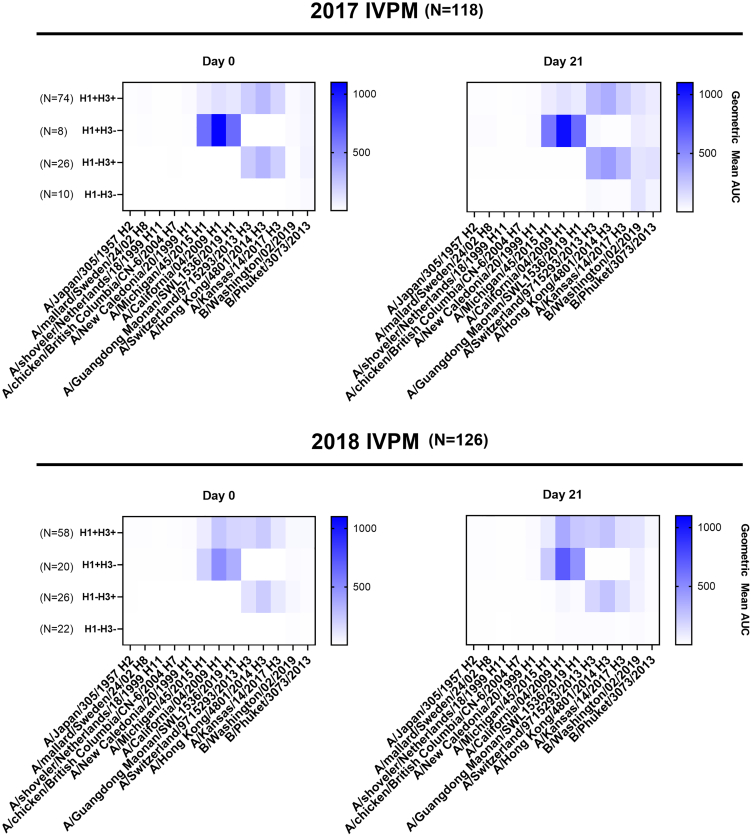
Breadth of serum antibody reactivity in children stratified by baseline reactivity Antibody levels against a panel of group 1, group 2 or influenza B hemagglutinins (HAs) are shown. 118 and 126 samples from children of the 2016–17 or 2017–18 seasons, respectively, were analyzed. Baseline reactivity prior to LAIV administration to group 1 (H1+), group 2 (H3+), or influenza B viruses was measured by the presence of serum antibodies determined using an influenza virus protein microarray (IVPM). Antibody levels are expressed as geometric mean (GM) area under the curve (AUC) values. The GM AUC for a determined group is represented by a single rectangle in the heatmap. Blank rectangles are indicative of a lack of reactivity. For statistical comparisons, refer to Table S3.

Baseline (pre-LAIV) reactivity to group 1 (H1+) or group 2 (H3+) influenza viruses was categorized by the presence of serum antibodies to a panel of H1 and H3 HA proteins in an influenza virus protein microarray (IVPM).^18^ While there are no data on circulating influenza viruses in The Gambia during the lifetime of the recruited children, data from neighboring Senegal suggested that the included HA constructs were suitable to capture prior influenza virus exposure (Figure S1).^19^ Among children recruited in 2017, 63% were H1+H3+ at baseline, with 7% H1+ alone, 22% H3+ alone and 8% seronegative for both H1 and H3 (Figure 1, Table S1), demonstrating high levels of influenza A virus exposure in early life. In the 2018 cohort, 46% were H1+H3+ at baseline, with 16% positive for H1 alone, 21% positive for H3 alone, and 17% seronegative for both.

Baseline vs. day 21 antibody levels suggested an increment in the overall magnitude and breadth of the anti-HA antibody response following vaccination (Figure 1). A proportion of double-negative (H1-H3-) or single positive (H1+H3- or H1+H3+) children turned double-positive (H1+H3+) following vaccination, yielding 65.3% double-positives in 2017 and 69.8% in 2018 (Table S1).

### Vaccination induces stalk-reactive antibodies with antibody dependent cellular cytotoxicity reporter activity

Broadly reactive antibodies are directed toward conserved regions of the influenza virus surface glycoproteins. The stalk domain of the HA is highly conserved among different subtypes of the same phylogenetic group.^20^ We previously reported that updating the LAIV pH1N1 component in 2018 improved immunogenicity as measured by hemagglutinin inhibition (HAI) assays CD4^+^ T cell responses, and may overcome the poor efficacy and effectiveness reported in previous years.^16^ We therefore evaluated the induction of anti-stalk antibodies by the administration of both the 2017 and 2018 LAIV formulations. For this, we used chimeric HA constructs displaying an exotic head domain (to which no to little pre-existing immunity is expected in humans) and the stalk of group 1 (cH6/1) or group 2 (cH7/3) HA. H1 HA IVPM responses and group 1 stalk ELISAs correlated well, as did H3 HA IVPM responses and group 2 stalk ELISAs (Spearman correlation coefficients 0.54–0.60, Figure S2). However, not all H1 or H3 positive individuals in IVPM had detectable stalk responses, and some individuals unreactive in the IVPM assay had detectable stalk responses by ELISA; likely due to differences in the sensitivity of the assays (Figure S2). Similar correlation was observed when comparisons were made with HAI measured in a previous study (Figure S3).^16^

**Figure 2:**
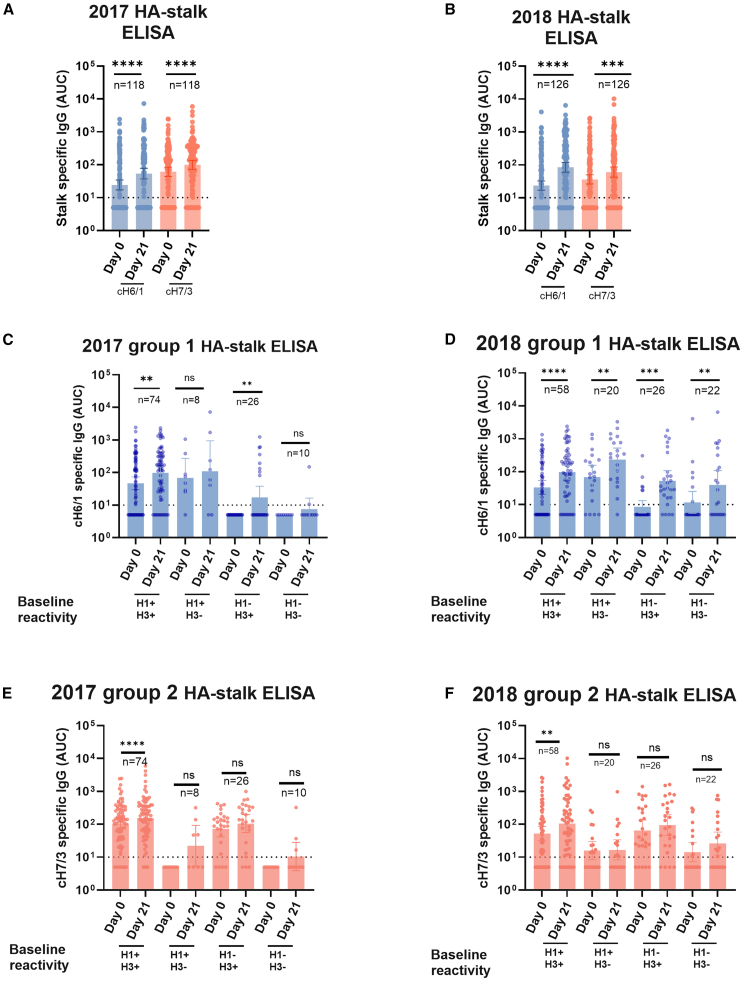
Serum induction of stalk reactive antibodies by LAIV administration in children (A and B) Group 1 or group 2 stalk reactive antibodies were measured using chimeric hemagglutinins bearing the stalk domain of group 1 (cH6/1) or group 2 (cH7/3) influenza viruses. Samples from 118 to 126 children from the 2016-17 (left column) and 2017-18 (right column) season were analyzed. Baseline and post-vaccination (day 21) antibody levels are shown in (A) and (B). (C–F) Samples were stratified by infection exposure based on an influenza virus protein microarray (IVPM): baseline and post-vaccination antibodies against group 1 (C and D) or group 2 (E and F) stalk are shown. Bars represent the geometric mean AUC pre and post vaccination for every age group, and error bars indicate the 95% confidence interval. The horizontal dotted lines indicate the assay limit of detection (LoD); values below this threshold were assigned half the LoD. Statistical comparisons were performed using a Wilcoxon matched-paired signed-rank test: *p* < 0.05 was considered statistically significant with a 95% confidence level. Statistical differences between baseline and post vaccination levels are shown. ∗*p* ≤ 0.05, ∗∗*p* ≤ 0.01, ∗∗∗*p* ≤ 0.001, ∗∗∗∗*p* ≤ 0.0001. Numbers on top of every pair of bars indicate sample size.

We detected a significant increase of both group 1 and group 2 stalk-reactive antibodies after vaccination in both 2017 and 2018 (Figures 2A and 2B respectively), including the boosting of pre-existing titers in children with evidence of prior exposure to H1 and H3 viruses (Figures 2C–2F). Of note, the highest antibody induction was detected in 2018 against group 1 stalk, where children received NY15 containing LAIV (Figure 2B), which is consistent with our previously reported HAI titer increases from the same study.^16^ Overall, the induction/boosting of group 1 stalk reactive antibodies was observed more commonly than group 2 across different baseline HA-seroreactive categories, with several children who were H1-H3+ at baseline inducing a group 1 stalk response.

**Figure 3:**
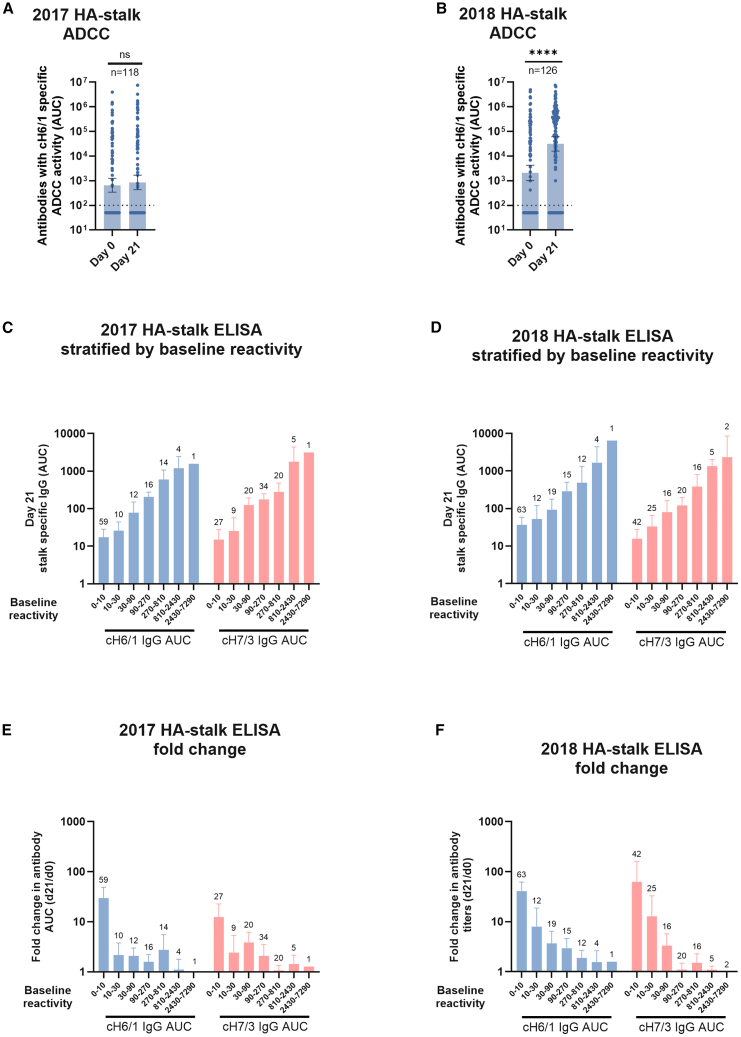
Serum stalk reactive antibodies induced by LAIV administration display ADCC activity (A and B) Group 1 stalk reactive antibodies with ADCC activity were measured using an ADCC reporter assay in Madin-Darby canine kidney (MDCK) cells stably expressing the cH6/1 antigen on the surface. Baseline and post-vaccination antibody levels with ADCC activity to cH6/1 are shown in (A) and (B), respectively. (C–F) In (C–F), group 1 or group 2 stalk reactive antibodies were measured using chimeric hemagglutinins bearing the stalk domain of group 1 (cH6/1) or group 2 (cH7/3). Samples from participants were stratified by baseline reactivity (indicated in the X axis). Stalk reactive IgG levels induced by vaccination, expressed as area under the curve (AUC), are shown in (C) and (D) for the 2016–17 and 2017–18, respectively. Fold change in stalk reactive IgG, calculated as antibody levels at day 21 post-vaccination divided by baseline levels (d21/d0) is shown in E and F for the 2016–17 and 2017–18 seasons, respectively. Bars represent the geometric mean AUC (A–D) or geometric mean fold change (E–F). Error bars indicate the 95% confidence interval. Statistical comparisons were performed using a Kruskal-Wallis test corrected for multiple comparisons with Dunn’s post-test: *p* < 0.05 was considered statistically significant with a 95% confidence level. ∗∗∗∗*p ≤* 0.0001*,* ns = not significant. Numbers on top of every pair of bars indicate sample size. The horizontal dotted lines in A and B indicate the assay limit of detection (LoD); values below this threshold were assigned half the LoD.

Antibodies directed against the HA stalk are prone to trigger cellular effector functions that contribute to virus clearance and protection *in vivo.*^21^^,^^22^ To assess the effector function capacity of stalk-reactive antibodies induced by vaccination, we used an antibody dependent cellular cytotoxicity (ADCC) reporter assay in which Madin-Darby canine kidney (MDCK) cells stably expressing the cH6/1 chimeric HA are incubated with serum dilutions, followed by the addition of the effector reporter cells.^23^ Strikingly, stalk-reactive antibodies induced by the 2018 formulation displayed strong effector functions (Figure 3B), but antibodies from vaccinees of 2017 displayed little to no ADCC reporter activity (Figure 3A). Regardless of baseline reactivity, stalk-reactive antibodies induced by the 2018 vaccine displayed ADCC reporter activity (Figure S4). Overall, these findings indicate not only that the LAIV formulation from 2018 was able to induce higher titers of stalk-reactive antibodies but also that these antibodies display effector functions *in vitro*.

Significant increases in serum stalk-reactive antibodies following LAIV were seen more commonly in younger age groups (Figure S5). However, multivariable linear regression models demonstrated that baseline stalk antibody levels (*p* < 0.0001 for both group 1 and group 2 stalks) and vaccine formulation for group 1 stalk (*p* = 0.0228), but not age, were predictors of day 21 stalk antibody levels (Figure S6). While the fold-change from baseline anti-stalk antibodies was greater in individuals with low baseline titers, the absolute increases in these groups were modest. Thus, day 21 titers were still higher in individuals who had higher pre-existing stalk-specific titers from influenza virus exposure (Figures 3C–3F).

**Figure 4:**
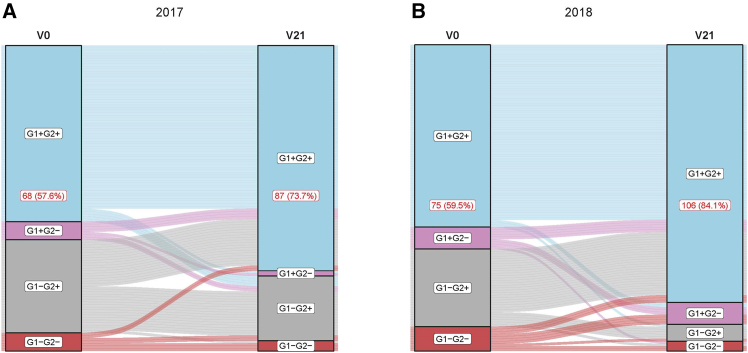
Changes in baseline seropositivity after LAIV administration Sankey plots depict changes from baseline stalk seropositivity measured by ELISA. Individuals were categorized based on their seropositivity profile to group 1 (G1+) or group 2 (G2+) hemagglutinin (HA) stalk: G1-G2-, G1-G2+, G2+G1-, or G1+G2+. Baseline (V0, to the left) and Day 21 post LAIV (V21, to the right) are shown. The links between nodes depict the proportion of changes from Baseline to Day 21 in the different reactivity groups.

Regardless of these low titers observed, seroconversion to both group 1 and group 2 stalk was induced by LAIV. The proportion of children reactive to both group 1 and 2 (G1+G2+) increased from 57.6% to 73.7% in 2017 and 59.5% to 84.1% in 2018 (Figure 4, Table S2), demonstrating the capacity of LAIV to induce a *de novo* stalk response to one group in the context of pre-existing immunity to another. The induction of antibodies to both group 1 and group 2 simultaneously was modest in children who were seronegative to both prior to LAIV receipt. In 2017, 2 of 7 G1-G2- children seroconverted to become G1+G2+, with 3 of 10 G1-G2- children seroconverting to G1+G2+ in 2018.

### Vaccination induced poor anti-neuraminidase antibody levels in serum and mucosal antibodies in oral fluid

**Figure 5:**
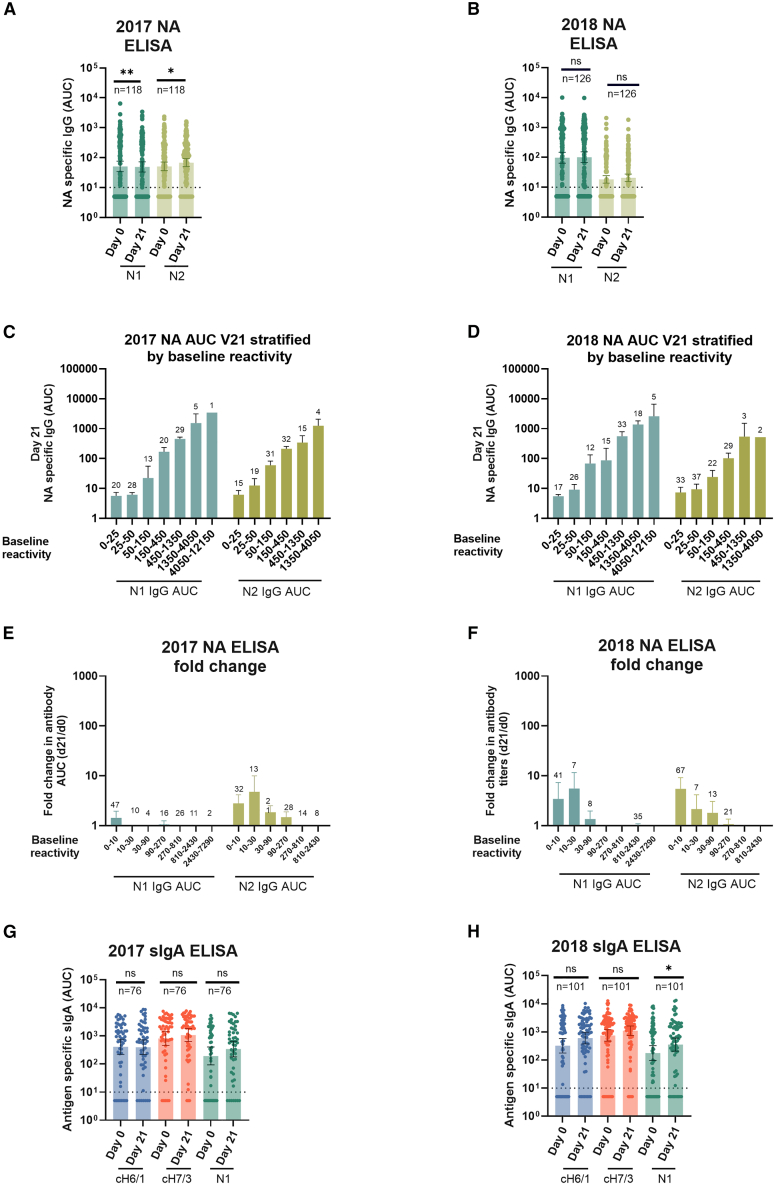
Serum NA reactive antibodies and mucosal secretory IgA (sIgA) induced by vaccination (A and B) Group 1 or group 2 neuraminidase (NA) reactive antibodies were measured using recombinant N1 or N2 proteins. Baseline and post-vaccination antibody levels in samples from 118 to 126 children from the 2016–17 and 2017–18 seasons, respectively, are shown in (A) and (B), respectively. (C and D) In C and D samples from participants were stratified by baseline reactivity (indicated in the X axis). (E and F) In (E) and (F), fold change in NA reactive IgG was calculated as the levels at day 21 post-vaccination divided by baseline levels (d21/d0) for the 2016-17 (E) and 2017-18 (F) seasons respectively. (G and H) In (G–H), sIgA mucosal antibodies in oral fluid were measured against N1 neuraminidase, and cH6/1 or cH7/1 chimeric hemagglutinins (HAs). Baseline and post-vaccination antibody levels are shown. In A–B and G–H, the horizontal dotted lines indicate the assay limit of detection (LoD); values below this threshold were assigned half the LoD. Statistical comparisons were performed using a Wilcoxon matched-paired signed-rank test (A–B, G–H). *p* < 0.05 is considered statistically significant with a 95% confidence level. Statistical differences between baseline and post vaccination levels are shown. ns = not significant, ∗*p* ≤ 0.05, ∗∗*p* ≤ 0.01. Numbers on top of every pair of bars indicate sample size.

Antibodies against the surface glycoprotein neuraminidase can block viral infection and prevent virus transmission.^24^^,^^25^ Moreover, mucosal antibody responses, especially secretory IgA (sIgA), can prevent infection and transmission at local mucosal sites.^26^ However, current influenza virus vaccines induce poor serum anti-neuraminidase antibodies and mucosal responses in adults.^27^ We determined if a single dose of LAIV at early stages in life was able to improve the induction of these responses. Following LAIV administration, serum anti-N1 and N2 antibody induction was minimal (Figures 5A and 5B), regardless of baseline antibody levels or baseline H1/H3 reactivity (Figures 5C–5F, and Figure S7). However, stratification by baseline reactivity confirmed that children positive to H1 displayed antibodies against N1 and those positive to H3 had N2-reactive antibodies (Figure S7). Similar to stalk-reactive antibodies, the fold-change from baseline was greater in children with low baseline titers, but the absolute increases in these groups were modest, being Day 21 titers still higher in children with higher pre-existing stalk-specific antibodies derived from influenza virus exposure. Stratification by baseline H1/H3 reactivity did not show patterns of neuraminidase or mucosal antibody induction following vaccination. We also assessed the induction of anti-stalk and NA sIgA titers in oral fluid. Only low induction of stalk-reactive or NA-reactive sIgA was detected (Figures 5G and 5H). In addition, sIgA patterns observed (pre- and post-vaccination) did not follow the same baseline H1/H3 reactivity (Figure S8). Overall, such as adults, data indicates that LAIV-vaccinated children elicit poor anti-neuraminidase antibodies and mucosal sIgA.

## Discussion

There is evidence to suggest that influenza virus infections early in life leave an imprint on immunological memory that shapes future encounters with influenza viruses and influenza vaccines. A hallmark article by Gostic and colleagues in 2016 suggested that, based on epidemiological data, individuals first infected with group 1 HA expressing viruses (H1N1, H2N2) were better protected from severe outcomes during zoonotic infections with H5N1 (group 1 HA).^2^ Individuals infected first with H3N2 (group 2) were found to be better protected against severe outcomes with H7N9 zoonotic infections (also group 2 HA).^2^ Similar observations have been made in animal models.^23^ It was speculated that this effect may be due to the imprinting of immunological memory to either the conserved group 1 HA stalk or the conserved group 2 HA stalk. This potential imprinting effect may also have an impact on vaccine effectiveness later in life, especially when considering novel, stalk-based vaccines.

In November 2019, a meeting was convened by the Bill and Melinda Gates Foundation in Seattle to discuss if and how imprinting could be optimized and used in favor of broad protection. Stalk-directed antibodies have been shown to be protective in animal models and are a possible correlate of protection in humans.^6^^,^^10^^,^^28^^,^^29^ One hypothesis was that vaccination with a trivalent LAIV at a very young age could either leave a balanced stalk-imprint (and potentially NA imprint) in the immune memory of naive subjects or could perhaps balance out biased imprinting that already occurred.^7^ Using samples from LAIV studies in The Gambia, we set out to investigate anti-stalk and anti-NA responses pre- and post-LAIV in children aged 24–59 months in two consecutive seasons in which the H1N1 component of the LAIV differed.

We found that baseline reactivity per se to H1N1 or H3N2 does not necessarily determine if LAIV induces an anti-stalk response, and LAIV was able to boost pre-existing stalk titers. The overall induction of anti-stalk immunity was, however low, and induction of anti-NA immunity and secretory IgA to both stalk and NA by LAIV was negligible. However, LAIV was able to induce seroconversion to both group 1 and 2 stalks, regardless of the prior immunity, resulting in an overall increase in the number of children with both group 1 and 2 stalk reactive antibodies. This in effect resulted in the equalizing of anti-stalk immunity, although the levels of new stalk reactivity were low compared to those induced by prior influenza exposure. Despite the cohort consisting of influenza vaccine naive children, only a small proportion were seronegative to both H1 and H3 HA (or both group 1 and group 2 stalk). This limited the ability to explore our original hypothesis that LAIV could imprint immunity to both groups simultaneously. While we did observe some children who seroconverted to both groups, this only occurred in approximately 30% of children. Whether a 2^nd^ dose of LAIV, as is often given to influenza vaccine naive children, could further enhance stalk reactive antibodies and/or induce simultaneous seroconversion in more children would be important to assess in future studies. Antibodies to the H1 stalk induced by LAIV also showed increased ADCC activity as measured by a reporter assay. Importantly, Fc-FcR mediated effector functions for stalk-reactive antibodies have been connected to protection *in vivo*, at least in animal models.^30^

Another finding was the difference between the two vaccine formulations. In previous work, we described that updating the H1N1 component from A/17/California/2009/38 (Cal09) used in the 2016-17 season to A/17/New York/15/5364 (NY15) used in the 2017-18 formulation, improved vaccine shedding and immunogenicity, as determined by serum hemagglutination inhibition titers and CD4^+^ T cell responses.^16^ These findings stressed the importance of replicative fitness besides antigenicity during the annual selection of vaccine components. Here, we found that the 2017-18 formulation overall elicited higher titers of group 1 stalk-reactive antibodies that displayed strong effector functions as measured by an ADCC reporter assay. Hence, these immunological readouts provide partial mechanistic support of the higher efficacy observed using the 2017-18 formulation.

In summary, we show that LAIV is able to boost pre-existing stalk antibodies and induce modest titers in children who are naive to either group 1 or 2 influenza A viruses. Future studies are needed to determine if LAIV can lead to balanced imprinting or not.

### Limitations of the study

Our study also has limitations worth considering. Firstly, only children above 24 months of age were enrolled due to the current licensing and WHO pre-qualification status of LAIV. Although a proportion (8.6%) of children were not reactive to both H1N1 and H3N2 at this age, most children were seropositive against one or more antigens from group 1, group 2 or B influenza viruses. The ideal scenario to evaluate the potential of LAIV as the prime antigenic exposure would be in seronegative children below 2 years, and even more suitably below 1 year of age. Future studies exploring this and assessing the immune responses elicited by secondary exposures to vaccination or to infection following a first LAIV exposure would be essential. Moreover, assessing the durability of such responses and their effectiveness at preventing influenza virus infections, reducing severe disease and hospitalization, and preventing death would be key to determining if an equivalent imprinting effect is achievable. Another limitation is that we were only able to measure antibody responses. Antibodies are produced by two types of B-cells: plasmablasts and long-lived plasma cells. However, B-cell immunological memory is based on memory B-cells. They patrol the periphery and can differentiate in plasmablasts quickly when encountering an antigen, but do not produce antibodies. Therefore, our measurements of antibodies are just a proxy, but not a direct measurement of immune memory. While the stalk-specific antibody titers induced by LAIV may be low, memory B-cells may be elicited in the stalk-seroconverters that provide protective immune responses following future exposures. Finally, due to the sample collection capacity at the study site, we only assessed sIgA in oral fluid and not in nasal secretions. Antibody titers in nasal secretions are generally higher as compared to oral fluid, and the nasal cavity is the port of entry for influenza viruses. Notwithstanding, although the magnitude of the response is lower in oral fluid, titers in both types of fluids correlate well.^31^

## Resource availability

### Lead contact

Further information and requests for resources and reagents should be directed to and will be fulfilled by the lead contact, Florian Krammer (florian.krammer@mssm.edu).

### Materials availability

Unique reagents used in the study are available upon request from the lead author.

### Data and code availability

• Data: All data will be available from Figshare under the following https://doi.org/10.6084/m9.figshare.28848047.
• Code: No code was used or developed for this study.
• Any additional information required to reanalyze the data reported in this article is available from the lead contact upon request.

## Acknowledgments

We would like to thank Hisaaki Kawabata for technical assistance. The study was partially supported by the 10.13039/100000865Bill and Melinda Gates Foundation (INV-004222, T.I.d.S.), the Wellcome Trust (110058/Z/15/Z, T.I.d.S), and 10.13039/100000060NIAID grant R21 AI151917 (F.K.). For the purpose of Open Access, the author has applied a CC BY publication copyright licence to any Author Accepted Manuscript version arising from this submission. Work on the revision for this article was supported by NIAID Centers of Excellence for Influenza Research and Response (CEIRR, 75N93021C00014) and Collaborative Influenza Vaccine Innovation Centers (CIVICs, 75N93019C00051). We would like to thank the study participants and their parents who took part in the study and the field and nursing staff led by Dr. Edwin Armitage, Janko Camara and Sulayman Bah.

## Author contributions

F.K., J.M.C., and T.I.D.S. conceptualized the study; P.M., K.S., J.T., A.R., G.S., M.F., M.L., B.F., and D.B. performed experiments; Y.J.J. and H.J.S. managed biospecimen processing and biobanking; J.M.C., F.S., and T.I.D.S. analyzed data; F.K., J.M.C., and T.I.D.S. administered the project; F.K. and T.I.D.S. provided resources; and J.M.C., F.K., and T.I.D.S. wrote the original draft. All authors reviewed, edited, and approved the final version of the article and have had access to the raw data. J.M.C., F.K., and T.I.D.S. can verify the accuracy of the raw data for the study.

## Declaration of interests

The Icahn School of Medicine at Mount Sinai has filed patent applications regarding influenza virus vaccines on which FK is listed as an inventor. The Krammer laboratory has received support for influenza virus research in the past from GSK and is currently receiving support from Dynavax. FK is currently consulting for GSK, Third Rock Ventures, Pfizer, and Avimex.

## STAR★Methods

### Key resources table

**Table:** 

REAGENT or RESOURCE	SOURCE	IDENTIFIER
**Antibodies**		

anti-Human IgG (Fc specific)-Peroxidase antibody produced in goat	Sigma-Aldrich	Cat#A0170; RRID: AB_257868
mouse anti-human secretory IgA	MilliporeSigma	Cat#411423; RRID: AB_212059
goat anti-mouse IgG Fc antibody	Thermo Fisher Scientific	Cat#31439; RRID: AB_228292
HA stalk-reactive monoclonal antibody CR9114	Krammer laboratory at the Icahn School of Medicine at Mount Sinai	Reagents | Krammer Laboratory (mssm.edu)
Cy5-labeled anti-human IgG secondary antibody	Abcam	Cat#ab97172

**Bacterial and virus strains**		

A/Japan/305/1957 (H2N2)	Krammer laboratory at the Icahn School of Medicine at Mount Sinai	Reagents | Krammer Laboratory (mssm.edu)
A/mallard/Sweden/24/02 (H8N4)	Krammer laboratory at the Icahn School of Medicine at Mount Sinai	Reagents | Krammer Laboratory (mssm.edu)
A/shoveler/Netherlands/18/1999 (H11N7)	Krammer laboratory at the Icahn School of Medicine at Mount Sinai	Reagents | Krammer Laboratory (mssm.edu)
A/chicken/British Columbia/CN-6/2004 (H7N3)	Krammer laboratory at the Icahn School of Medicine at Mount Sinai	Reagents | Krammer Laboratory (mssm.edu)
A/New Caledonia/20/1999 (H1N1)	Krammer laboratory at the Icahn School of Medicine at Mount Sinai	Reagents | Krammer Laboratory (mssm.edu)
A/Michigan/45/2015 (H1N1)	Krammer laboratory at the Icahn School of Medicine at Mount Sinai	Reagents | Krammer Laboratory (mssm.edu)
A/California/04/2009 (H1N1)	Krammer laboratory at the Icahn School of Medicine at Mount Sinai	Reagents | Krammer Laboratory (mssm.edu)
A/Guangdong Maonan/SWL1536/2019 (H1N1)	Krammer laboratory at the Icahn School of Medicine at Mount Sinai	Reagents | Krammer Laboratory (mssm.edu)
A/Switzerland/9715293/2013 (H3N2)	Krammer laboratory at the Icahn School of Medicine at Mount Sinai	Reagents | Krammer Laboratory (mssm.edu)
A/Hong Kong/4801/2014 (H3N2)	Krammer laboratory at the Icahn School of Medicine at Mount Sinai	Reagents | Krammer Laboratory (mssm.edu)
A/Kansas/14/2017 (H3N2)	Krammer laboratory at the Icahn School of Medicine at Mount Sinai	Reagents | Krammer Laboratory (mssm.edu)
B/Washington/02/2019 (B/Victoria/2/87-like lineage)	Krammer laboratory at the Icahn School of Medicine at Mount Sinai	Reagents | Krammer Laboratory (mssm.edu)
B/Phuket/3073/2013 (B/Yamagata/16/88-like lineage)	Krammer laboratory at the Icahn School of Medicine at Mount Sinai	Reagents | Krammer Laboratory (mssm.edu)

**Biological samples**		

Whole blood from 2 timepoints (Day 0 and Day 21 post vaccination)	Study in Sukuta, in The Gambia	ClinicalTrials.gov (NCT02972957)
Oral fluids from 2 timepoints (Day 0 and Day 21 post vaccination)	Study in Sukuta, in The Gambia	ClinicalTrials.gov (NCT02972957)

**Chemicals, peptides, and recombinant proteins**		

A/Michigan/45/2015 pH1N1	Krammer laboratory at the Icahn School of Medicine at Mount Sinai	https://labs.icahn.mssm.edu/krammerlab/reagents/
B/Washington/02/2019 (B/Victoria/2/87-like lineage)	Krammer laboratory at the Icahn School of Medicine at Mount Sinai	https://labs.icahn.mssm.edu/krammerlab/reagents/
A/mallard/Sweden/81/02 (H6N1)	Krammer laboratory at the Icahn School of Medicine at Mount Sinai	https://labs.icahn.mssm.edu/krammerlab/reagents/
A/Puerto Rico/08/34 (H1N1)	Krammer laboratory at the Icahn School of Medicine at Mount Sinai	https://labs.icahn.mssm.edu/krammerlab/reagents/
A/Anhui/1/13 (H7N9)	Krammer laboratory at the Icahn School of Medicine at Mount Sinai	https://labs.icahn.mssm.edu/krammerlab/reagents/
A/Perth/16/09 (H3N2)	Krammer laboratory at the Icahn School of Medicine at Mount Sinai	https://labs.icahn.mssm.edu/krammerlab/reagents/
A/Michigan/45/2015 (H1N1)	Krammer laboratory at the Icahn School of Medicine at Mount Sinai	https://labs.icahn.mssm.edu/krammerlab/reagents/
A/Hong Kong/4801/2014 (H3N2)	Krammer laboratory at the Icahn School of Medicine at Mount Sinai	https://labs.icahn.mssm.edu/krammerlab/reagents/
Quick Start™ Bradford 1x Dye Reagent	Bio-Rad	Cat#5000205

**Critical commercial assays**		

Clear Flat-Bottom Immulon 4 HBX 96-Well Plates	Thermo Fisher	Cat#3855
Bio-Glo™ Luciferase Assay System	Promega	Cat#G7940

**Deposited data**		

Data underlying the figures	This paper	Figshare: https://doi.org/10.6084/m9.figshare.28848047

**Experimental models: Cell lines**		

MDCK Cells	Krammer laboratory at the Icahn School of Medicine at Mount Sinai	https://labs.icahn.mssm.edu/krammerlab/reagents/

**Software and algorithms**		

Prism 9	GraphPad	https://www.graphpad.com/
Packages tidyverse (v2.0) and ggplot2 (v3.4.2)	R and R studio	https://www.r-project.org/

### Experimental model and study participant details

Samples were obtained from an open-label, prospective, observational, phase 4 immunogenicity study in Sukuta, a peri-urban area in The Gambia, previously described in detail by Lindsey et al.^16^ Data from this study correspond to all children enrolled in the randomized trial that received the LAIV. Eligibility comprised children of 24–59 months of age, clinically well, and with no history of respiratory illness within the past 14 days. Complete study criteria can be found in ClinicalTrials.gov (NCT02972957). The current study was approved by The Gambia Government and UK Medical Research Council (MRC) joint ethics committee and the Medicines Control Agency of The Gambia. The study was performed according to International Conference on Harmonisation Good Clinical Practice standards. Signed or thumb printed informed consent of parents from the children participating was obtained.

118 children received one dose of the Cal09 LAIV from 2016 to 17 and a different cohort of 126 children received one dose of the NY15 LAIV from 2017 to 18.

Samples were collected on day 0 (baseline) and day 21 after vaccine administration. Whole blood was obtained for serum separation. Oral fluids were obtained by passive absorption using swabs placed in between gums and buccal mucosa (ORACOL+, Malvern Medical Development, Worcester, UK). Serum samples and oral fluid were stored at −70°C before further processing. Nasovac-S - Influenza Vaccine (Human, Live Attenuated, Serum Institute of India Pvt. Ltd) was used.

#### Recombinant proteins

All recombinant proteins were expressed in High Five insect cells and purified from cell culture supernatants as previously described.^32^ Recombinant HA proteins used in this study were derived from the following isolates: A/Japan/305/1957 (H2N2), A/mallard/Sweden/24/02 (H8N4), A/shoveler/Netherlands/18/1999 (H11N7), A/chicken/British Columbia/CN-6/2004 (H7N3), A/New Caledonia/20/1999 (H1N1), A/Michigan/45/2015 (H1N1), A/California/04/2009 (H1N1), A/Guangdong Maonan/SWL1536/2019 (H1N1), A/Switzerland/9715293/2013 (H3N2), A/Hong Kong/4801/2014 (H3N2), A/Kansas/14/2017 (H3N2), B/Washington/02/2019 (B/Victoria/2/87-like lineage) and B/Phuket/3073/2013 (B/Yamagata/16/88-like lineage). As a proxy to measure the antibody response against the vaccine strains A/17/New York/15/5364 pH1N1 and B/Texas/02/2013 (B/Victoria/2/87-like lineage), we used recombinant HA from A/Michigan/45/2015 pH1N1 and B/Washington/02/2019 (B/Victoria/2/87-like lineage). To measure HA-stalk reactive antibodies by ELISA, we used chimeric HA proteins (cHA) expressing an avian head domain and the stalk domain of either H1 or H3.^33^ The protein carrying a group 1 stalk, namely cH6/1, bears the head domain of A/mallard/Sweden/81/02 (H6N1) and the stalk domain of A/Puerto Rico/08/34 (H1N1). The group 2 stalk protein cH7/3 displays the head domain of A/Anhui/1/13 (H7N9) and the stalk domain of A/Perth/16/09 (H3N2). Recombinant NA proteins used in ELISA were derived from the following isolates: A/Michigan/45/2015 (H1N1) and A/Hong Kong/4801/2014 (H3N2). The amino acid sequence homology between the H1 from pH1N1 strains is 99.3% and between the B HAs is 99.1%. Briefly, cultures were infected with recombinant baculoviruses at a multiplicity of infection (MOI) of 10. Cell culture supernatants were then harvested by low-speed centrifugation 72 h post infection and were purified using Ni2+-nitrilotriacetic acid (Ni-NTA) chromatography.^32^^,^^34^ Protein purity and identity were tested by sodium dodecyl-sulfate polyacrylamide gel electrophoresis (SDS-PAGE) and Coomassie staining. Final protein concentrations were determined with Bradford reagent.

### Method details

#### Serum ELISA

96-well microtiter plates (Immulon 4 HBX; Thermo Fisher) were coated with the corresponding chimeric hemagglutinin (cHA) or neuraminidase (NA) proteins at a concentration of 2 μg/mL overnight at 4°C. Plates were then washed the next day three times with phosphate-buffered saline (PBS) containing 0.1% Tween 20 (PBS-T). Blocking solution containing PBS-T, 3% goat serum and 0.5% milk powder was added to the plates (200 μL/well) and incubated for 1 h at 20°C. Blocking solution was removed, samples were serially diluted 3-fold and added to the plates at a starting dilution of 1:100 in blocking solution (100 μL/well). Plates were incubated for 2 h at 20°C, then washed three times with PBS-T. The secondary anti-Human IgG (Fc specific)-Peroxidase antibody produced in goat (Sigma-Aldrich) was added at a volume of 50 μL/well, incubating for 1 h at 20°C. Plates were washed four times with PBS-T and developed with SigmaFast *o*-phenylenediamine dihydrochloride (OPD; Sigma) for 10 min at 20°C, then the reaction was stopped with 3M hydrochloric acid (Thermo Fisher Scientific). Using a Synergy H1 microplate reader (BioTek), the plates were read at an optical density (OD) of 490 nm. Background level was calculated as the average plus three times the standard deviation of blank wells in which no sample was added. Antibody levels expressed as the area under the curve (AUC) were calculated using Prism 9 (GraphPad).

#### Antigen specific oral fluid sIgA ELISA

Total IgA values were available as previously described, with data generated using an ELISA and sample dilutions of 1:1000 to 1:20000.^35^ To measure antigen-specific sIgA, Immulon 4 HBX 96-well microtiter plates (Thermo Fisher Scientific) were coated overnight at 4°C with recombinant proteins (100 ng/well) in PBS (pH 7.4). Well contents were discarded and blocked with 200 μL of 5% non-fat milk (AmericanBio) in PBS containing 0.1% Tween 20 (PBS-T) for one hour at RT. After blocking, 50 μL of oral fluid samples diluted with 2.5% non-fat milk in PBST were added to each well. Oral fluid samples were diluted to IgA concentrations of either 10 (for samples with total IgA concentrations between 10 and 15 mg/mL), 15 (for samples with total IgA concentrations between 15 and 20mg/mL), or 20 (for samples with total IgA concentrations above 20 mg/mL) mg/mL based on the total IgA concentration of each sample (described in previous section), and then serially diluted 2-fold. Plates containing samples were incubated overnight at 4°C. After washing with PBS-T three times, 50 μL of mouse anti-human secretory IgA antibody (MilliporeSigma, #411423) diluted to 5 μg/mL with 2.5% non-fat milk in PBS-T was added to each well, and incubated at RT for 2 h. These plates were washed again with PBS-T three times, and 50 μL of horse-radish peroxidase (HRP) labeled goat anti-mouse IgG Fc antibody (Thermo Fisher Scientific, #31439) diluted to 1:1000 with 2.5% non-fat milk in PBS-T was added to each well, and incubated at RT for 1 h. After washing with PBS-T three times, 100 μL of SIGMAFAST *o*-phenylenediamine dihydrochloride substrate solution (Sigma-Aldrich) was added to each well for 10 min at RT. Reaction was stopped by addition of 50 μL of 3M hydrochloric acid (Thermo Fisher Scientific). Optical density at 490 nm was measured using Synergy 4 (BioTek) plate reader. Eight blank wells were used to assess background and AUC was calculated by subtracting the average of blank values plus three times standard deviation of the blank values. Antigen specific sIgA AUC values were adjusted by dividing the values by the IgA concentration of oral fluid samples (either 10, 15, or 20) for analysis.

#### Antibody-mediated cellular cytotoxicity (ADCC) reporter assay

ADCC reporter assays were performed according to the kit manufacturer’s instructions (Promega) with minor modifications and similar to assays described by us earlier.^23^ Madin-Darby canine kidney (MDCK) cells stably expressing the chimeric HA cH6/1 antigen (described in ‘recombinant protein expression’ section) were seeded in Dulbecco’s Modified Eagle Medium (DMEM), 3.0x10^4^ cells/well were seeded onto white flat-bottom 96-well plates (Costar). The next day, serum dilutions were prepared starting at 1:50 initial dilution followed by 3-fold dilutions in assay buffer consisting of Roswell Park Memorial Institute (RPMI) medium supplemented with L-glutamine and 0.5% ultra-low IgG fetal bovine serum (FBS). As a positive control, the HA stalk-reactive monoclonal antibody CR9114^36^ was used at an initial dilution of 10μg/mL in assay buffer. Cell culture media from MDCK cell plates was aspirated, and monolayers were washed once with PBS (Gibco). RPMI 1640 medium (Gibco) (25 μL) and pre-diluted sera (25μL) were added into each of the corresponding wells. Reporter cells, specifically Jurkat cells expressing the Fc FcɣRIIIa, (Promega) were added into the wells at a concentration of 7 ×10^4^/well (50μL). The plates were incubated in a cell culture incubator for six hours at 37°C with 5% CO_2_. After the incubation time, Bio-Glo™ Assay Reagent (Promega) (75μL) was added and plates were read immediately using the Synergy H1 microplate reader (BioTek). The plates were read at a wavelength of 125 nm, and the file was exported into Excel.

#### Influenza virus protein microarrays (IVPM)

IVPMs were produced by printing recombinant influenza virus HAs onto expoxysilane-coated glass slides (Schott, Mainz, Germany).^18^^,^^37^ The protein panel used was selected based on coverage of representative HAs from: a) group 1 influenza A virus representative HAs (H1, H2, H8 and H11), b) group 2 influenza A virus representative HAs (H3 and H7), and c) influenza B virus HAs from the B/Victoria/2/87 and B/Yamagata/16/88 lineages. Each slide contains 24 arrays comprised of 13 HAs diluted in 0.1% milk PBS and printed in triplicate at a volume of 30 nL per spot at a concentration of 100 μg/mL. All IVPMs were vacuum-packed after printing and stored at −80°C until use. Before use, IVPM slides were allowed to warm to room temperature, then incubated in a humidity chamber which was maintained at 95–98% relative humidity for 2 h in order to bind proteins to the slide and inactivate any expoxysilane residues not in contact with the printed recombinant HAs. After binding, IVPM slides were placed into 96-well microarray gaskets (Arrayit, Sunnyvale, CA, USA), physically dividing each slide into 24 separate arrays for the assay. The arrays were then blocked with 220 μL 3% milk PBS containing 0.1% Tween 20 (PBS-T) for 2 h. Afterward, blocking solution was removed from the arrays and serum samples diluted 1:100 in 1% milk PBS-T were incubated with the arrays at a volume of 100 μL, serially diluted 1:10 across three arrays. The sera were then removed from the arrays and the arrays were washed 3 times with 220 μL PBS-T. After washing, 100 μL of Cy5-labeled anti-human IgG secondary antibody diluted 1:3000 in 1% milk PBS-T was added and incubated for 1 h. The secondary antibody solution was then removed and each array was washed another 3 times with PBS-T. After washing, slides were removed from their gaskets and rinsed with PBS-T and deionized water before being dried with an air compressor. Arrays were imaged with a Vidia microarray scanner (Indevr, Boulder, CO, USA) using an exposure time of 1000 ms. Area under the curve was calculated from the median spot fluorescence, taking the total peak area with a minimum threshold of 0.04. The assay cutoff was defined previously and was determined based on reactivity of antibody depleted sera.^18^ This cutoff was used to determine baseline reactivity to H1 or H3 recombinant proteins.

### Quantification and statistical analysis

A Wilcoxon matched-paired signed-rank test was used for statistical comparisons of AUC values between pre and post vaccination. A Kruskal-Wallis test corrected for multiple comparisons with Dunn’s post-test was used was used across different groups. All *p* values less than 0.05 were considered statistically significant with a 95% confidence interval. Statistical analyses were performed with Prism 9 (GraphPad, USA).

### Additional resources

Complete study criteria can be found in ClinicalTrials.gov (NCT02972957).
