# Supplementary material for: Induction of hemagglutinin stalk reactive antibodies by the administration of a live-attenuated influenza virus vaccine in children
Source: iScience. 2025 Jun 13;28(7):112893. doi: 10.1016/j.isci.2025.112893 (PMC12226393; doi:10.1016/j.isci.2025.112893)
Supplement: Document S1. Figures S1–S8 and Tables S1–S3 [file mmc1.pdf]

iScience, Volume *28*

Supplemental information

Induction of hemagglutinin stalk reactive antibodies by the administration of a live-attenuated influenza virus vaccine in children

Juan Manuel Carreño, Philip Meade, Na Fatimata Sogodogo, Kaori Sano, Johnstone Tcheou, Ariel Raskin, Gagandeep Singh, Miriam Fried, Madhumathi Loganathan, Benjamin Francis, Dominika Bielak, Ya Jankey Jagne, Hadijatou J. Salah, Florian Krammer, and Thushan I. de Silva

Supplementary Figure 1

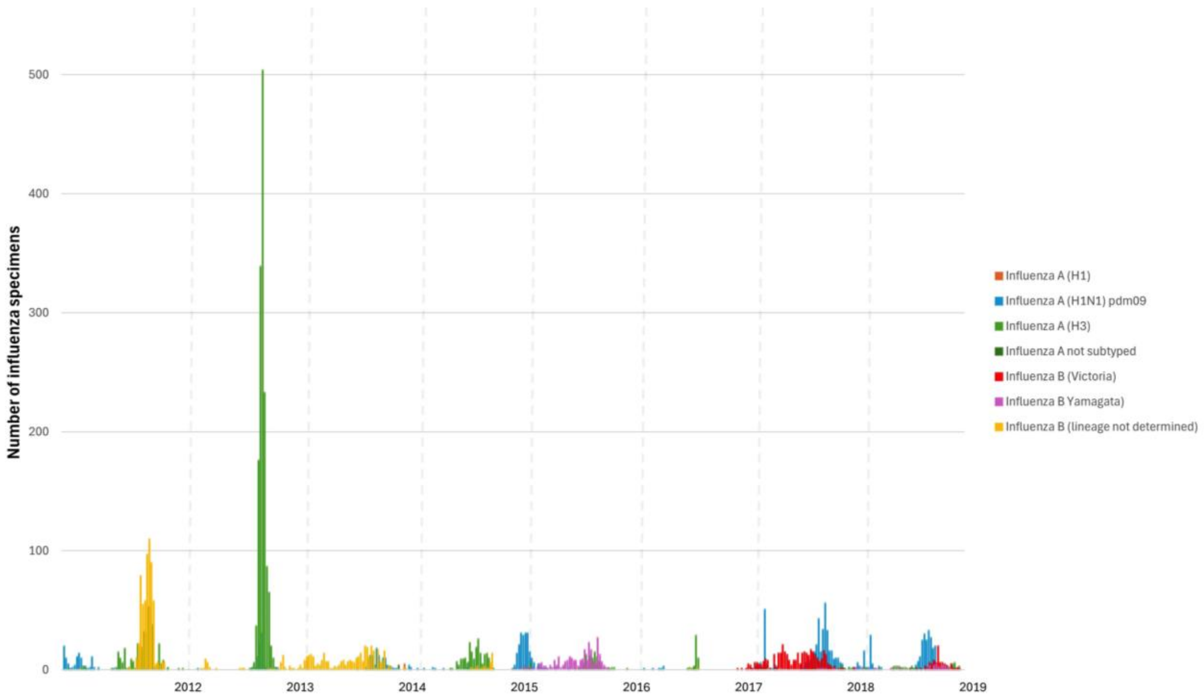

Supplementary figure 1. Influenza virus circulation in Senegal between 2011-2018. Influenza laboratory surveillance data obtained from the Global Influenza Surveillance and Response System (GISRS) of the World Health Organization (WHO). The number of influenza positive specimens of the corresponding subtypes are shown throughout every influenza season from 2011 to 2018. The source of the data is not indicated by the reporting country and may include sentinel or non-sentinel surveillance sources or both.

Supplementary Figure 2

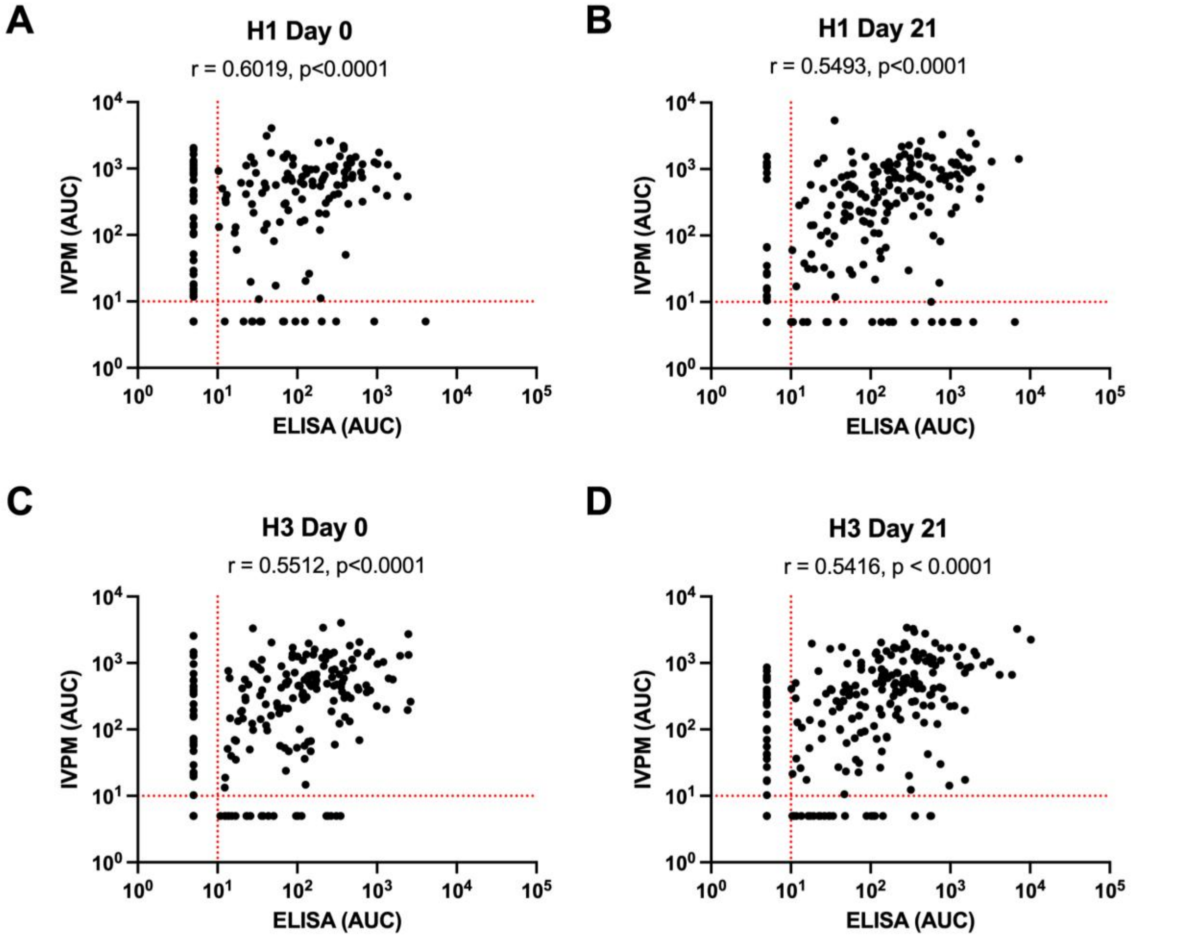

Supplementary figure 2. Correlation of antibody levels measured by ELISA and IVPM. The correlation between group 1 (H1) or group 2 (H3) stalk-reactive antibodies measured by an enzyme linked immunosorbent assay (ELISA) and full-length HA (H1 or H3) reactive antibodies measured by an influenza virus protein microarray (IVPM) was assessed. A-B, H1. C-D, H3. A, C, Baseline. B, D, day 21 post vaccination. AUC = Area Under the Curve. Spearman correlation coefficient is shown. P (two-tailed) < 0.0001.

Supplementary Figure 3

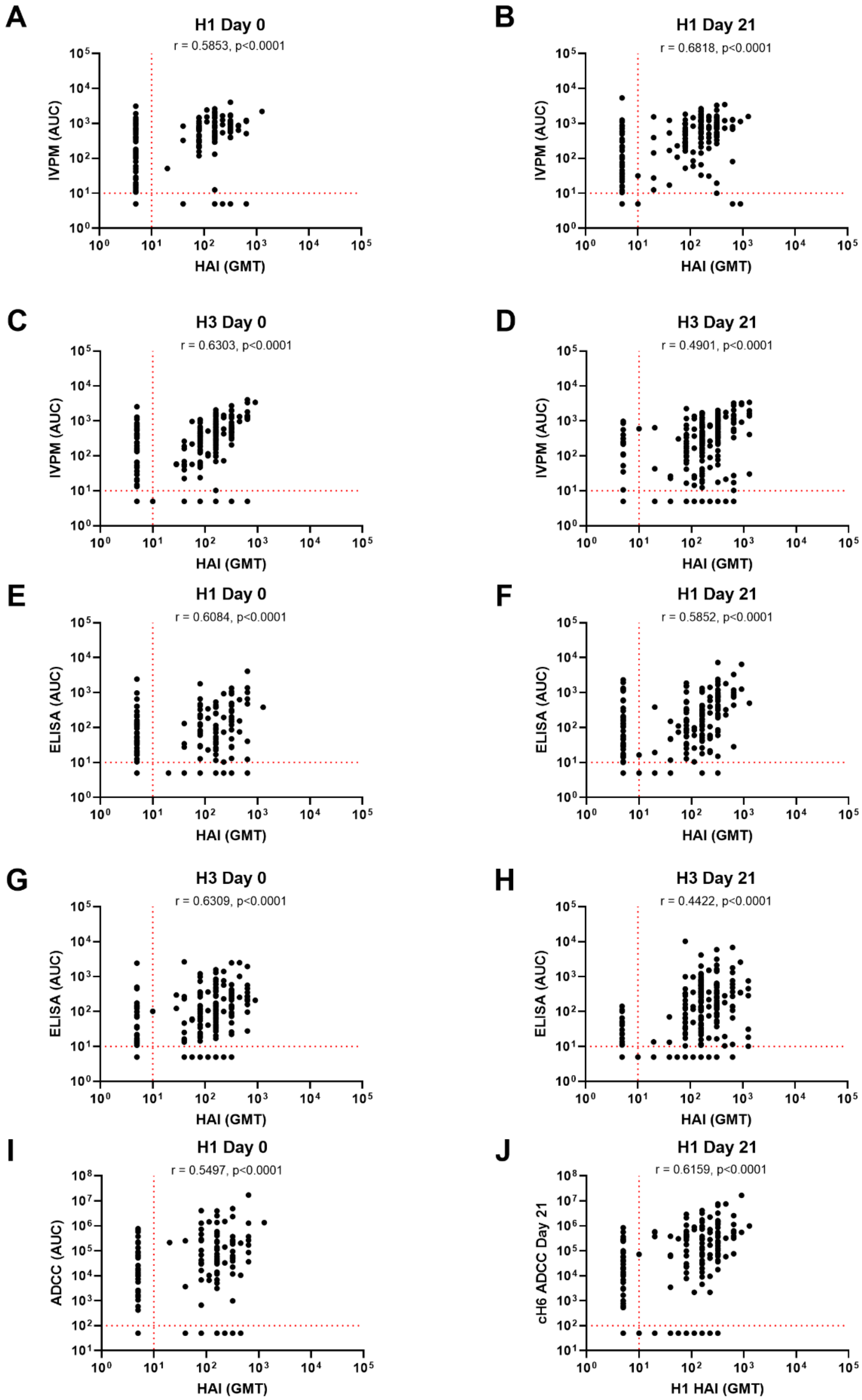

Supplementary Figure 3. Correlation of antibody levels between HAI and multiple assays (IVPM, ELISA, and ADCC). The correlation between group 1 (H1) and group 2 (H3) reactive antibodies measured by an hemagglutination inhibition assay (HAI), and reactive antibodies measured by IVPM (A – D), ELISA (E – H), and ADCC (I-J). AUC = Area Under the Curve. Spearman correlation coefficient is shown. P (two-tailed) < 0.0001.

Supplementary Figure 4

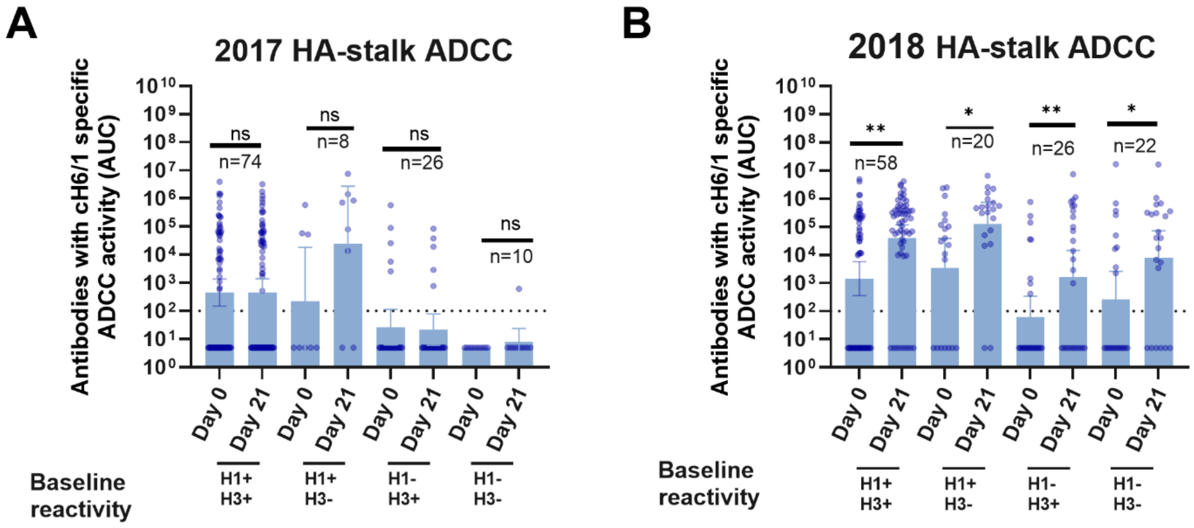

Supplementary figure 4. Serum stalk reactive antibodies with antibody dependent cellular cytotoxicity (ADCC) activity stratified by exposure. Group 1 stalk reactive antibodies with ADCC activity were measured using an ADCC reporter assay in Madin-Darby canine kidney (MDCK) cells stably expressing the cH6/1 antigen on the surface. Samples were stratified by infection exposure based on an influenza virus protein microarray (IVPM). Baseline and post-vaccination antibodies with cH6/1 specific ADCC activity were measured in 118 and 135 children from the 2016-17 and 2017-18 seasons respectively, and are shown in A and B. Bars represent the geometric mean AUC pre and post vaccination for every age group, and error bars indicate the 95% confidence interval. The horizontal dotted lines indicate the assay limit of detection (LoD); values below this threshold were assigned half the LoD. Statistical comparisons were performed using a Wilcoxon matched-paired signed-rank test: $P<0.05$ considered statistically significant with a 95% confidence level. Statistical differences between baseline and post vaccination levels are shown. *$P \leq 0.05$, **$P \leq 0.01$, ***$P \leq 0.001$, ****$P \leq 0.0001$. Numbers on top of every pair of bars indicate sample size.

Supplementary Figure 5

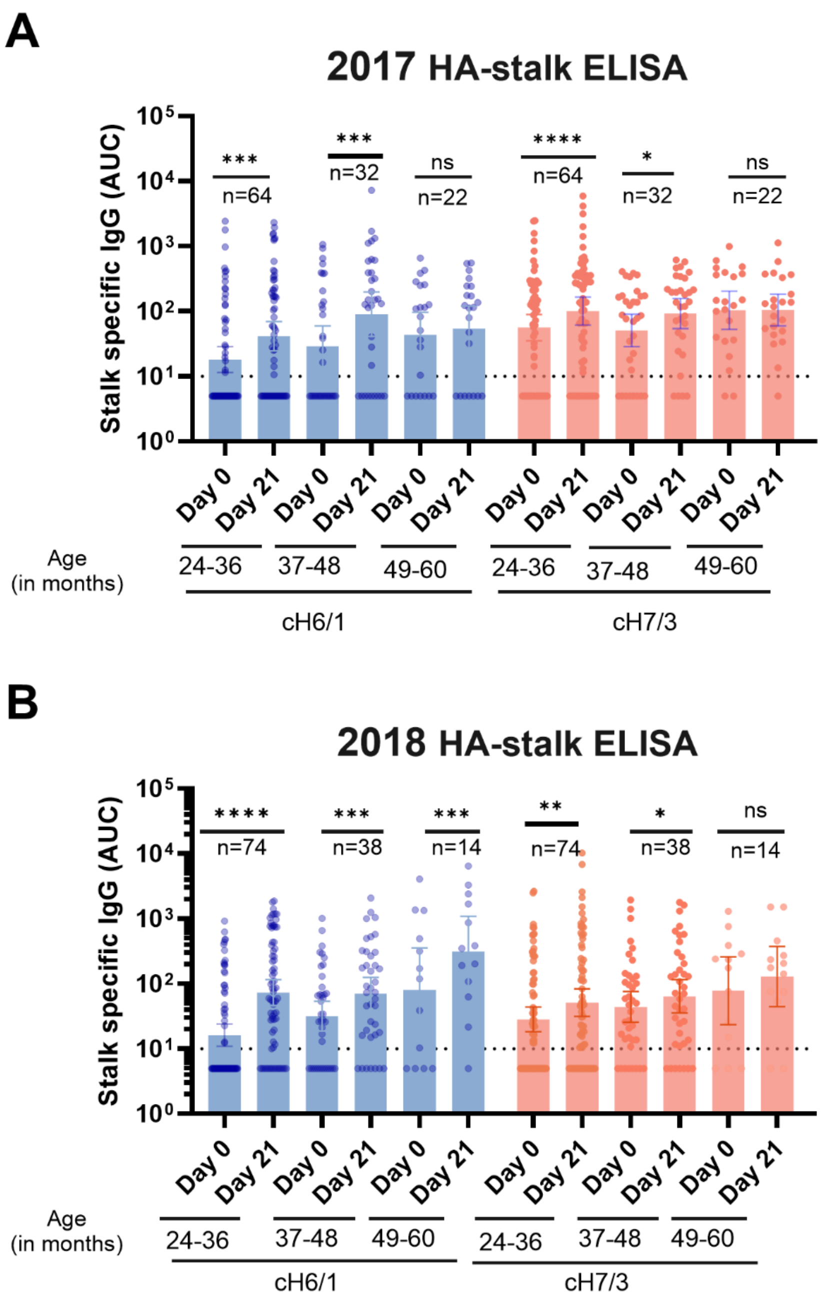

Supplementary figure 5. Stratification of serum stalk reactive antibodies by age. Group 1 or group 2 stalk reactive antibodies were measured using chimeric hemagglutinins bearing the stalk domain of group 1 (cH6/1) or group 2 (cH7/3). Baseline and post-vaccination antibody levels stratified by age of 118 and 135 children from the 2016-17 and 2017-18 season are shown in A and B respectively. Age stratification ranges included 24-36-, 37-48- and 49–59- month-old children. Bars represent the geometric mean AUC pre and post vaccination for every age group, and error bars indicate the 95% confidence interval. The horizontal dotted lines indicate the assay

limit of detection (LoD); values below this threshold were assigned half the LoD. Statistical comparisons were performed using a Wilcoxon matched-paired signed-rank test: $P<0.05$ considered statistically significant with a 95% confidence level. Statistical differences between baseline and post vaccination levels are shown. *$P \leq 0.05$, **$P \leq 0.01$, ***$P \leq 0.001$, ****$P \leq 0.0001$. Numbers on top of every pair of bars indicate sample size.

Supplementary Figure 6

A

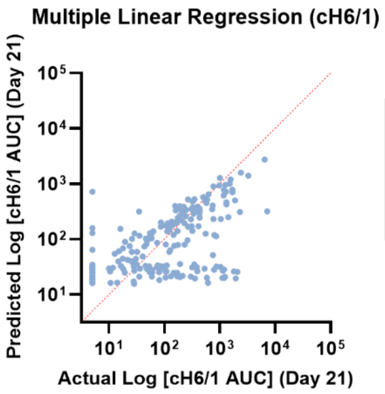

y = 0.71 + 0.68 (cH6/1 AUC Day 0) + 0.001 (Age) + 0.20 (Vaccine Year)

Variable	Estimate	95% CI (asymptotic)	P value
Intercept	0.7067	0.4661 to 0.9473	<0.0001
Log [cH6/1 AUC Day 0]	0.6804	0.5730 to 0.7877	<0.0001
Age (Months)	0.001332	-0.001113 to 0.003778	0.2843
Vaccine Year[2018]	0.2003	0.02812 to 0.3726	0.0228

B

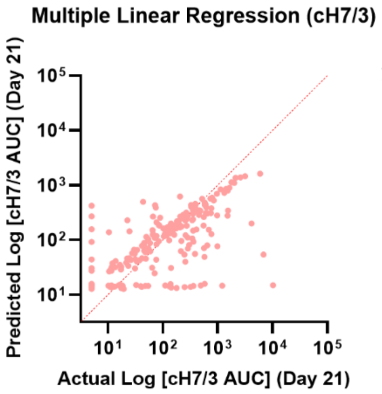

y = 0.69 + 0.75 (cH7/3 AUC Day 0) - 0.0006 (Age) - 0.03 (Vaccine Year)

Variable	Estimate	95% CI (asymptotic)	P value
Intercept	0.6880	0.4598 to 0.9163	<0.0001
Log [cH7/3 AUC Day 0]	0.7455	0.6501 to 0.8410	<0.0001
Age (Months)	-0.0006463	-0.002749 to 0.001456	0.5454
Vaccine Year[2018]	-0.02776	-0.1770 to 0.1215	0.7143

Supplementary figure 6. Multivariate analysis of age, baseline antibody titer, and vaccine year. A multiple linear regression was performed to assess the contribution of age, baseline titer, and vaccine year in the prediction of post-vaccination titer for group 1 and group 2 stalk antibodies (based on cH6/1 and cH7/3 reactivity). AUC values were log transformed before performing the multiple linear regression. For group 1, the multiple linear regression model is: y = 0.71 + 0.68 (cH6/1 AUC Day 0) + 0.001 (Age) + 0.20 (Vaccine Year), where y is the predicted AUC value post vaccination (Day 21). For group 2, the multiple linear regression model is: y = 0.69 + 0.75 (cH7/3 AUC Day 0) - 0.0006 (Age) - 0.03 (Vaccine Year). Where y is the predicted AUC value post vaccination (Day 21). In both groups, the regression model is statistically significant with a P<0.0001 with a 95% confidence interval. The asymptotic confidence interval is valid when the sample size is large.

Supplementary Figure 7

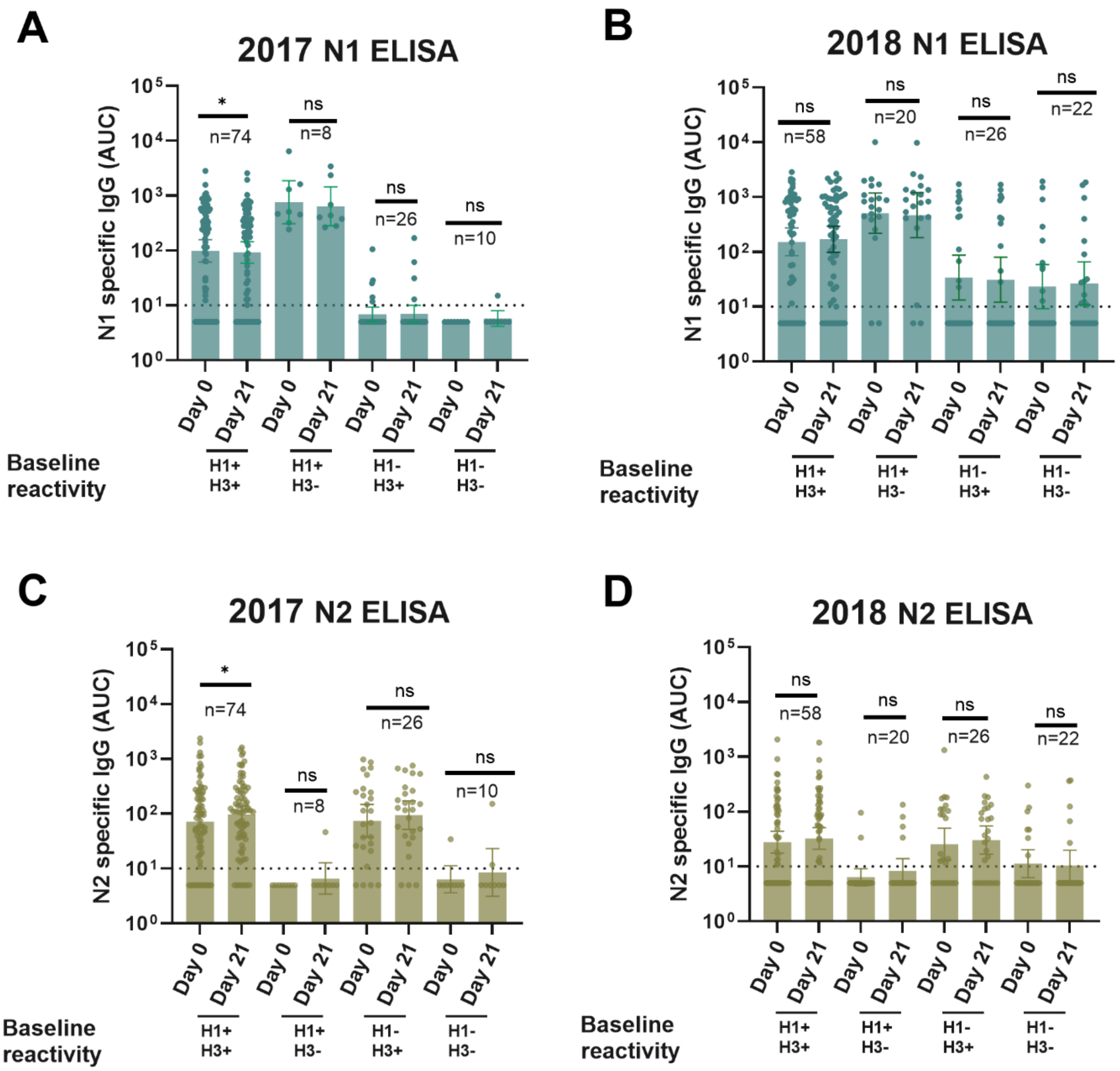

Supplementary figure 7. Stratification of serum NA reactive antibodies by exposure. Group 1 or group 2 neuraminidase (NA) reactive antibodies were measured using recombinant N1 or N2 proteins. Samples were stratified by infection exposure based on an influenza virus protein microarray (IVPM). Baseline and post-vaccination antibody levels of 118 and 135 children from the 2016-17 and 2017-18 season are shown in A and B for N1 reactive antibodies and in C and D for N2 reactive antibodies respectively. Bars represent the geometric mean AUC pre and post vaccination for every age group, and error bars indicate the 95% confidence interval. The horizontal dotted lines indicate the assay limit of detection (LoD); values below this threshold were assigned half the LoD. Statistical comparisons were performed using a Wilcoxon matched-paired signed-rank test: $P<0.05$ considered statistically significant with a 95% confidence level. Statistical differences between baseline and post vaccination levels are shown. *$P \leq 0.05$, **$P \leq 0.01$, ***$P \leq 0.001$, ****$P \leq 0.0001$. Numbers on top of every pair of bars indicate sample size

Supplementary Figure 8

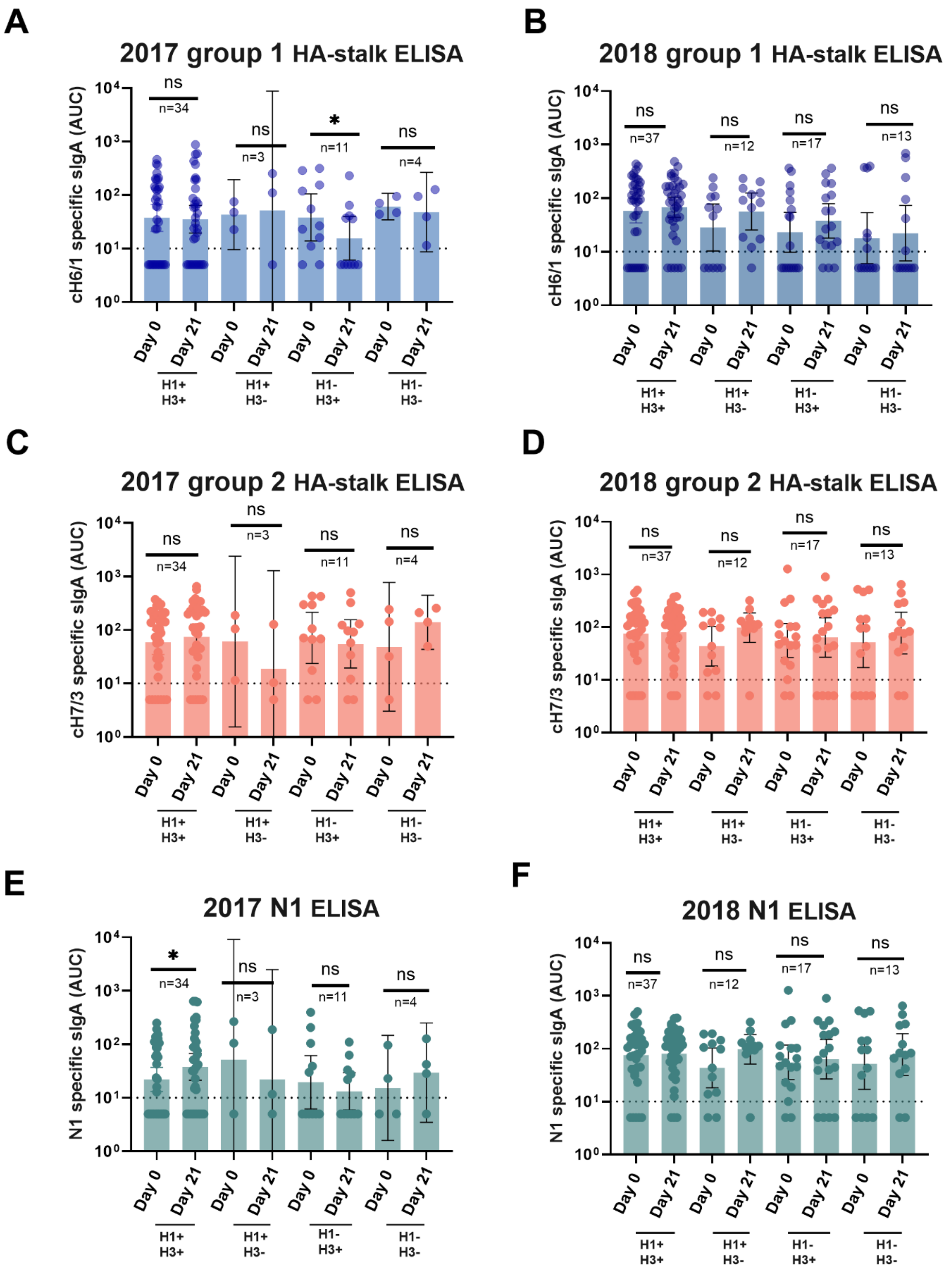

Supplementary figure 8. Mucosal secretory IgA (sIgA) antibodies stratified by exposure. sIgA mucosal antibodies were measured in oral fluid against N1 neuraminidase, and cH6/1 or cH7/1 chimeric hemagglutinins (HAs). Samples were stratified by infection exposure based on an influenza virus protein microarray (IVPM). Baseline and post-vaccination antibody levels of 118 and 135 children from the 2016-17 and 2017-18 respectively, are shown in A and B for group 1 stalk, C and D for group 2 stalk and E and F for N1. Bars represent the geometric mean AUC pre and post vaccination for every age group, and error bars indicate the 95% confidence interval. The horizontal dotted lines indicate the assay limit of detection (LoD); values below this threshold were assigned half the LoD. Statistical comparisons were performed using a Wilcoxon matched-paired signed-rank test: $P<0.05$ considered statistically significant with a 95% confidence level. Statistical differences between baseline and post vaccination levels are shown. *$P \leq 0.05$, **$P \leq 0.01$, ***$P \leq 0.001$, ****$P \leq 0.0001$. Numbers on top of every pair of bars indicate sample size

Supplementary table 1. Seropositivity to group 1 and group 2 hemagglutinin (HA) at baseline and 21 days after LAIV administration - IVPM

	2017				2018			
Group	Day 0 (N)	Day 21 (N)	Day 0 (%)	Day 21 (%)	Day 0 (N)	Day 21 (N)	Day 0 (%)	Day 21 (%)
**G1+G2+**	74	77	62.7	65.3	58	88	46	69.8
**G1+G2-**	8	2	6.8	1.7	20	18	15.9	14.3
**G1-G2+**	26	33	22.0	27.9	26	12	20.6	9.5
**G1-G2-**	10	6	8.5	5.1	22	8	17.5	6.3

The percentage of individuals displaying antibodies against the viral hemagglutinin (HA) of group 1 (H1) or group 2 (H3) influenza viruses was estimated using data from an influenza virus protein microarray (IVPM) from samples collected before (Day 0) and after (Day 21) receipt of the corresponding live attenuated influenza vaccine (LAIV) in 2017 or 2018. Individuals were categorized based on their seropositivity profile to group 1 (G1+) or group 2 (G2+) full-length hemagglutinin (HA): G1-G2-, G1-G2+, G2+G1-, or G1+G2+.

Supplementary table 2. Seropositivity to group 1 and group 2 hemagglutinin (HA) stalk at baseline and 21 days after LAIV administration - ELISA

	2017				2018			
Group	Day 0 (N)	Day 21 (N)	Day 0 (%)	Day 21 (%)	Day 0 (N)	Day 21 (N)	Day 0 (%)	Day 21 (%)
**G1+G2+**	68	87	57.6	73.7	75	106	59.5	84.1
**G1+G2-**	7	2	5.9	1.7	9	9	7.1	7.1
**G1-G2+**	36	25	30.5	21.2	32	7	25.4	5.6
**G1-G2-**	7	4	5.9	3.4	10	4	7.9	3.2

The percentage of individuals displaying antibodies against the stalk domain from the viral hemagglutinin (HA) of group 1 (H1) or group 2 (H3) influenza viruses was estimated using ELISA data from samples collected before (Day 0) and after (Day 21) receipt of the corresponding live attenuated influenza vaccine (LAIV) in 2017 or 2018. Individuals were categorized based on their seropositivity profile to group 1 (G1+) or group 2 (G2+) hemagglutinin stalk: G1-G2-, G1-G2+, G2+G1-, or G1+G2+.

Supplementary Table 3. Statistics of data presented in Figure 1.

2017					
**Antigens**		**H1+H3+**	**H1+H3-**	**H1-H3+**	**H1-H3-**
**A/Japan/305/1957 H2**	Pre/Post AUC Comparison	ns p=0.2218	ns p=0.6250	ns p=0.0625	NA
	Pre/Post Fold Change	1.61	8.33	1.63	1.00
	Geometric Mean Pre	11.4 (SD 3.31 [8.62-15.0])	7.92 (SD 2.63 [3.53-17.8])	5.00 (SD 1.00 [5.00-5.00])	5.00 (SD 1.00 [5.00-5.00])
	Geometric Mean Post	12.2 (SD 3.71 [9.04-16.6])	16.1 (SD 3.84 [5.22-49.5)	6.37 (SD 1.76 [5.07-8.01])	5.00 (SD 1.00 [5.00-5.00])
**A/mallard/Sweden/24/02 H8**	Pre/Post AUC Comparison	ns p=0.4072	ns p=0.3125	ns p=0.2500	NA
	Pre/Post Fold Change	3.44	12.17	2.03	1.00
	Geometric Mean Pre	14.3 (SD 3.38 [10.8-19.0])	10.1 (SD 2.84 [4.23-24.2])	5.00 (SD 1.00 [5.00-5.00])	5.00 (SD 1.00 [5.00-5.00])
	Geometric Mean Post	13.0 (SD 3.86 [9.49-17.8])	17.4 (SD 6.28 [3.74-80.8])	6.37 (SD 2.07 [4.75-8.54])	5.00 (SD 1.00 [5.00-5.00])
**A/shoveler/Netherlands/18/1999 H11**	Pre/Post AUC Comparison	ns p=0.4742	ns p>0.9999	ns p>0.9999	NA
	Pre/Post Fold Change	2.50	1.36	1.24	1.00
	Geometric Mean Pre	8.65 (SD 2.99 [6.71-11.2])	6.01 (SD 1.68 [3.89-9.30])	5.00 (SD 1.00 [5.00-5.00])	5.00 (SD 1.00 [5.00-5.00])
	Geometric Mean Post	8.08 (SD 2.89 [6.32-10.3])	7.12 (SD 2.71 [3.09-16.4])	5.40 (SD 1.48 [4.61-6.31])	5.00 (SD 1.00 [5.00-5.00])
**A/chicken/British Columbia/CN-6/2004 H7**	Pre/Post AUC Comparison	ns p=0.9935	ns p>0.9999	ns p=0.8750	NA
	Pre/Post Fold Change	2.49	1.07	5.19	1.00
	Geometric Mean Pre	9.31 (SD 2.66 [7.42-11.7])	6.95 (SD 2.54 [3.19-15.1])	6.03 (SD 2.25 [4.35-8.37])	5.00 (SD 1.00 [5.00-5.00])
	Geometric Mean Post	9.38 (SD 3.01 [7.27-12.1])	5.59 (SD 1.37 [4.29-7.29])	6.95 (SD 2.70 [4.65-10.4])	5.00 (SD 1.00 [5.00-5.00])
**A/New Caledonia/20/1999 H1**	Pre/Post AUC Comparison	ns p=0.5958	ns p=0.0938	ns p=0.1250	NA
	Pre/Post Fold Change	1.89	3.47	2.32	1.00
	Geometric Mean Pre	22.6 (SD 3.38 [17.1-30.0])	11.4 (SD 3.24 [4.28-30.0])	5.00 (SD 1.00 [5.00-5.00])	5.00 (SD 1.00 [5.00-5.00])
	Geometric Mean Post	20.6 (SD 3.61 [15.3-27.7])	25.0 (SD 3.65 [8.46-73.7])	6.65 (SD 2.16 [4.87-9.07])	5.00 (SD 1.00 [5.00-5.00])
**A/Michigan/45/2015 H1**	Pre/Post AUC Comparison	ns p=0.6395	ns p=0.3828	ns p=0.1250	NA
	Pre/Post Fold Change	3.78	1.15	2.02	1.00
	Geometric Mean Pre	89.3 (SD 7.83 [55.5-144])	624 (SD 2.13 [331-1176])	5.00 (SD 1.00 [5.00-5.00])	5.00 (SD 1.00 [5.00-5.00])
	Geometric Mean Post	86.6 (SD 8.27 [53.1-141])	584 (SD 2.43 [278-1226])	6.73 (SD 2.08 [5.00-9.04])	5.00 (SD 1.00 [5.00-5.00])
**A/California/04/2009 H1**	Pre/Post AUC Comparison	ns p=0.6721	ns p=0.3125	ns p=0.1250	NA
	Pre/Post Fold Change	2.58	1.06	2.61	1.00
	Geometric Mean Pre	140 (SD 7.64 [87.1-224])	1087 (SD 1.64 [718-1646])	5.00 (SD 1.00 [5.00-5.00])	5.00 (SD 1.00 [5.00-5.00])
	Geometric Mean Post	133 (SD 8.66 [80.5-219])	1040 (SD 1.55 [722-1497])	7.17 (SD 2.41 [5.03-10.2])	5.00 (SD 1.00 [5.00-5.00])
**A/Guangdong Maonan/SWL1536/2019 H1**	Pre/Post AUC Comparison	ns p=0.6885	ns p=0.3828	ns p=0.1250	NA
	Pre/Post Fold Change	2.10	1.23	2.37	1.00
	Geometric Mean Pre	111 (SD 6.04 [73.0-168])	643 (SD 1.86 [383-1077])	5.00 (SD 1.00 [5.00-5.00])	5.00 (SD 1.00 [5.00-5.00])
	Geometric Mean Post	93.0 (SD 7.17 [58.9-147])	637 (SD 2.02 [354-1147])	7.04 (SD 2.30 [5.03-9.85])	5.00 (SD 1.00 [5.00-5.00])
**A/Switzerland/9715**	Pre/Post AUC Comparison	*** p=0.0007	* p=0.0156	ns p=0.0941	ns p=0.1250
	Pre/Post Fold Change	1.82	10.51	4.92	15.41

293/2013 H3	Geometric Mean Pre	223 (SD 3.51 [167-298])	5.00 (SD 1.00 [5.00-5.00])	218 (SD 5.17 [112-423])	5.00 (SD 1.00 [5.00-5.00])
	Geometric Mean Post	298 (SD 3.51 [223-399])	34.5 (SD 2.98 [13.9-85.9])	345 (SD 2.85 [226-527])	31.5 (SD 5.69 [6.31-157])
**A/Hong Kong/4801/2014 H3**	Pre/Post AUC Comparison	* p=0.0114	ns p=0.1250	ns p=0.4992	ns p=0.1250
	Pre/Post Fold Change	1.64	2.66	2.50	6.37
	Geometric Mean Pre	309 (SD 3.86 [226-422])	5.00 (SD 1.00 [5.00-5.00])	306 (SD 5.33 [156-602])	5.00 (SD 1.00 [5.00-5.00])
	Geometric Mean Post	366 (SD 4.18 [263-510])	9.45 (SD 2.24 [4.81-18.6])	414 (SD 2.88 [270-635])	18.5 (SD 3.51 [5.78-59.0])
**A/Kansas/14/2017 H3**	Pre/Post AUC Comparison	ns p=0.0818	ns p=0.1250	ns p=0.1814	ns p=0.1250
	Pre/Post Fold Change	2.64	5.24	4.06	7.17
	Geometric Mean Pre	186 (SD 3.75 [137-253])	5.00 (SD 1.00 [5.00-5.00])	199 (SD 4.95 [104-379])	5.00 (SD 1.00 [5.00-5.00])
	Geometric Mean Post	224 (SD 3.80 [165-306])	12.9 (SD 3.28 [4.78-34.8])	283 (SD 3.39 [173-463])	20.0 (SD 3.74 [5.92-67.7])
**B/Washington/02/2019**	Pre/Post AUC Comparison	**** p<0.0001	ns p=0.1562	**** p<0.0001	ns p=0.0625
	Pre/Post Fold Change	21.52	7.27	43.39	23.72
	Geometric Mean Pre	30.2 (SD 4.69 [21.1-43.2])	25.6 (SD 5.81 [5.89-112])	13.1 (SD 4.35 [7.21-23.7])	12.9 (SD 5.33 [2.76-60.8])
	Geometric Mean Post	127 (SD 5.06 [87.0-184])	83.1 (SD 4.64 [23.1-300])	119 (SD 7.04 [53.9-261])	125 (SD 7.83 [18.6-837])
**B/Phuket/3073/2013**	Pre/Post AUC Comparison	*** p=0.0003	ns p=0.4375	** p=0.0038	ns p=0.2500
	Pre/Post Fold Change	5.21	1.68	39.87	3.39
	Geometric Mean Pre	48.6 (SD 5.92 [32.2-73.4])	44.9 (SD 11.4 [5.87-343])	58.1 (SD 7.03 [26.4-128])	26.9 (SD 9.33 [3.40-212])
	Geometric Mean Post	83.4 (SD 8.05 [51.4-135])	65.6 (SD 12.2 [8.10-532])	138 (SD 9.85 [54.7-347])	52.9 (SD 21.0 [3.17-884])
**2018**					
**Antigens**		**H1+H3+**	**H1+H3-**	**H1-H3+**	**H1-H3-**
**A/Japan/305/1957 H2**	Pre/Post AUC Comparison	ns p=0.0764	* p=0.0117	ns p=0.1094	ns p>0.9999
	Pre/Post Fold Change	4.09	2.29	2.41	1.43
	Geometric Mean Pre	9.78 (SD 3.56 [7.00-13.7])	6.73 (SD 1.89 [5.00-9.06])	5.32 (SD 1.37 [4.68-6.05])	5.00 (SD 1.00 [5.00-5.00])
	Geometric Mean Post	13.1 (SD 3.98 [9.13-18.9])	11.2 (SD 2.77 [6.95-18.1])	7.49 (SD 2.28 [5.37-10.4])	5.57 (SD 1.64 [4.45-6.97])
**A/mallard/Sweden/24/02 H8**	Pre/Post AUC Comparison	ns p=0.9152	** p=0.0078	* p=0.0312	ns p=0.5000
	Pre/Post Fold Change	1.61	3.88	8.27	2.88
	Geometric Mean Pre	11.5 (SD 3.32 [8.39-15.8])	6.54 (SD 2.34 [4.39-9.74])	5.00 (SD 1.00 [5.00-5.00])	5.00 (SD 1.00 [5.00-5.00])
	Geometric Mean Post	12.2 (SD 3.74 [8.63-17.3])	12.8 (SD 3.36 [7.24-22.5])	8.85 (SD 3.66 [5.24-14.9])	6.28 (SD 2.26 [4.34-9.10])
**A/shoveler/Netherlands/18/1999 H11**	Pre/Post AUC Comparison	ns p=0.8536	ns p=0.3125	ns p=0.5000	NA
	Pre/Post Fold Change	1.68	3.03	1.26	1.00
	Geometric Mean Pre	6.82 (SD 2.44 [5.39-8.62])	6.27 (SD 1.89 [4.65-8.45])	5.00 (SD 1.00 [5.00-5.00])	5.00 (SD 1.00 [5.00-5.00])
	Geometric Mean Post	7.57 (SD 2.39 [6.02-9.52])	8.34 (SD 2.81 [5.14-13.5])	5.55 (SD 1.48 [4.73-6.51])	5.00 (SD 1.00 [5.00-5.00])
**A/chicken/British Columbia/CN-6/2004 H7**	Pre/Post AUC Comparison	ns p=0.4319	ns p>0.9999	** p=0.0039	ns p=0.5000
	Pre/Post Fold Change	1.05	1.73	3.90	1.54
	Geometric Mean Pre	9.68 (SD 2.87 [7.34-12.8])	6.45 (SD 2.19 [4.47-9.31])	5.00 (SD 1.00 [5.00-5.00])	5.00 (SD 1.00 [5.00-5.00])
	Geometric Mean Post	8.41 (SD 2.87 [6.38-11.1])	6.07 (SD 1.91 [4.48-8.22])	9.22 (SD 2.78 [6.10-13.9])	5.88 (SD 1.72 [4.59-7.54])
**A/New Caledoni**	Pre/Post AUC Comparison	* p=0.0497	* p=0.0419	** p=0.0039	ns p=0.2500

**a/20/1999 H1**	Pre/Post Fold Change	2.68	2.64	3.52	2.23
	Geometric Mean Pre	21.5 (SD 3.27 [15.7-29.3])	12.5 (SD 2.84 [7.68-20.4])	5.00 (SD 1.00 [5.00-5.00])	5.00 (SD 1.00 [5.00-5.00])
	Geometric Mean Post	26.0 (SD 3.77 [18.3-36.8])	18.8 (SD 3.97 [9.87-35.9])	8.86 (SD 2.58 [6.04-13.0])	6.59 (SD 2.13 [4.68-9.30])
**A/Michigan/45/2015 H1**	Pre/Post AUC Comparison	* p=0.0318	* p=0.0362	** p=0.0078	ns p=0.1250
	Pre/Post Fold Change	2.40	1.40	16.83	3.38
	Geometric Mean Pre	83.1 (SD 5.89 [52.1-132])	202 (SD 2.62 [129-318])	5.00 (SD 1.00 [5.00-5.00])	5.00 (SD 1.00 [5.00-5.00])
	Geometric Mean Post	98.7 (SD 6.42 [60.5-161])	232 (SD 3.20 [135-400])	11.7 (SD 4.57 [6.30-21.5])	7.35 (SD 2.51 [4.83-11.2])
**A/California/04/2009 H1**	Pre/Post AUC Comparison	* p=0.0141	* p=0.0328	**** p<0.0001	*** p=0.0010
	Pre/Post Fold Change	10.65	3.23	54.12	15.93
	Geometric Mean Pre	253 (SD 6.97 [152-422])	492 (SD 3.59 [271-896])	5.00 (SD 1.00 [5.00-5.00])	5.00 (SD 1.00 [5.00-5.00])
	Geometric Mean Post	370 (SD 5.29 [239-574])	711 (SD 1.99 [516-981])	40.5 (SD 7.71 [17.7-92.3])	19.2 (SD 4.87 [9.33-39.4])
**A/Guangdong Maonan/SWL1536/2019 H1**	Pre/Post AUC Comparison	ns p=0.0520	ns p=0.0637	*** p=0.0001	*** p=0.0010
	Pre/Post Fold Change	4.80	1.91	34.16	11.13
	Geometric Mean Pre	185 (SD 6.38 [113-301])	349 (SD 3.47 [195-626])	5.00 (SD 1.00 [5.00-5.00])	5.00 (SD 1.00 [5.00-5.00])
	Geometric Mean Post	241 (SD 5.38 [155-374])	472 (SD 2.32 [318-699])	25.0 (SD 6.59 [11.7-53.4])	17.2 (SD 4.13 [9.01-32.7])
**A/Switzerland/9715293/2013 H3**	Pre/Post AUC Comparison	ns p=0.2593	* p=0.0312	* p=0.0272	** p=0.0020
	Pre/Post Fold Change	1.68	3.24	2.71	7.68
	Geometric Mean Pre	176 (SD 4.93 [116-268])	5.00 (SD 1.00 [5.00-5.00])	129 (SD 3.35 [79.2-210])	5.00 (SD 1.00 [5.00-5.00])
	Geometric Mean Post	218 (SD 4.78 [145-329])	8.76 (SD 2.65 [5.56-13.8])	185 (SD 3.53 [111-308])	16.5 (SD 3.82 [8.97-30.4])
**A/Hong Kong/4801/2014 H3**	Pre/Post AUC Comparison	ns p=0.5846	ns p=0.1250	* p=0.2370	** p=0.0039
	Pre/Post Fold Change	1.67	4.67	3.25	6.07
	Geometric Mean Pre	230 (SD 5.73 [145-364])	5.00 (SD 1.00 [5.00-5.00])	224 (SD 3.67 [132-378])	5.00 (SD 1.00 [5.00-5.00])
	Geometric Mean Post	264 (SD 6.06 [164-424])	7.73 (SD 2.84 [4.74-12.6])	262 (SD 3.53 [158-437])	14.2 (SD 3.99 [7.56-26.7])
**A/Kansas/14/2017 H3**	Pre/Post AUC Comparison	ns p=0.5358	ns p=0.1250	* p=0.0393	** p=0.0078
	Pre/Post Fold Change	3.86	2.16	5.84	3.07
	Geometric Mean Pre	108 (SD 6.15 [66.7-173])	5.00 (SD 1.00 [5.00-5.00])	103 (SD 3.39 [63.1-169])	5.00 (SD 1.00 [5.00-5.00])
	Geometric Mean Post	134 (SD 5.60 [85.1-211])	7.18 (SD 2.16 [5.00-10.3])	139 (SD 4.12 [78.5-246])	10.9 (SD 2.65 [6.99-16.9])
**B/Washington/02/2019**	Pre/Post AUC Comparison	*** p=0.0010	ns p=0.0797	ns p=0.2290	* p=0.0137
	Pre/Post Fold Change	22.92	16.69	10.13	7.96
	Geometric Mean Pre	32.2 (SD 6.86 [19.8-54.4])	23.6 (SD 5.56 [10.6-52.7])	36.6 (SD 8.17 [15.7-85.6])	10.0 (SD 3.78 [5.38-18.7])
	Geometric Mean Post	123 (SD 5.28 [79.4-190])	73.3 (SD 5.24 [33.8-159])	61.6 (SD 6.88 [28.3-134])	24.7 (SD 6.07 [10.9-56.2])
**B/Phuket/3073/2013**	Pre/Post AUC Comparison	ns p=0.1969	ns p=0.7422	ns p=0.8501	* p=0.0312
	Pre/Post Fold Change	2.93	1.60	15.94	8.32
	Geometric Mean Pre	30.8 (SD 7.74 [18.0-52.8])	15.3 (SD 7.48 [5.97-39.2])	16.7 (SD 6.98 [7.61-36.6])	6.41 (SD 1.92 [4.73-8.70])
	Geometric Mean Post	41.0 (SD 8.42 [23.4-71.8])	18.8 (SD 6.89 [7.60-46.3])	20.5 (SD 7.51 [9.07-46.2])	(SD 5.16 [5.54-24.7)

Geometric mean AUC values are summarized for each exposure group.
AUC: Area under the curve; ns: not significant; Pre: Geometric mean AUC prior to vaccination; Post: Geometric mean AUC post vaccination. Listed ranges represent Lower and Upper 95% CI of geometric mean.
For statistical comparisons between pre and post vaccination AUC values a Wilcoxon matched-pairs signed-rank test was used. $P<0.05$ considered statistically significant with a 95% confidence level. Statistical differences between baseline and post vaccination levels are shown. *$P \leq 0.05$, **$P \leq 0.01$, ***$P \leq 0.001$, ****$P \leq 0.0001$. Fold change after vaccine is presented (Post/Pre).
